# Calix[4]arene Polyamine Triazoles: Synthesis, Aggregation and DNA Binding

**DOI:** 10.3390/ijms232314889

**Published:** 2022-11-28

**Authors:** Vladimir Burilov, Egor Makarov, Diana Mironova, Elza Sultanova, Islamiya Bilyukova, Kevser Akyol, Vladimir Evtugyn, Daut Islamov, Konstantin Usachev, Timur Mukhametzyanov, Svetlana Solovieva, Igor Antipin

**Affiliations:** 1 Alexander Butlerov Institute of Chemistry, Kazan Federal University, 18 Kremlevskaya Str., 420008 Kazan, Russia; 2 Interdisciplinary Center for Analytical Microscopy, Kazan Federal University, 18 Kremlevskaya Str., 420008 Kazan, Russia; 3 Laboratory for Structural Studies of Biomacromolecules, FRC Kazan Scientific Center of RAS, 2/31 Lobachevskogo Str., 420111 Kazan, Russia; 4 Arbuzov Institute of Organic and Physical Chemistry, FRC Kazan Scientific Center of RAS, 8 Arbuzov Str., 420088 Kazan, Russia

**Keywords:** calixarene, click chemistry, polyamines, DNA binding, amphiphiles

## Abstract

Artificial gene delivery systems are in great demand from both scientific and practical biomedical points of view. In this paper, we present the synthesis of a new click chemistry calix[4]arene precursor with free lower rim and new water-soluble calixarene triazoles with 12 amino-groups on the upper rim (one with free phenol hydroxyl groups and two another containing four butyl or tetradecyl fragments). Aggregation in the series of amino-triazole calixarenes of different lipophilicity (calixarene with free phenol hydroxyl groups or butyl and tetradecyl fragments on the lower rim) was studied using dynamic light scattering and fluorescent pyrene probe. It was found that calix[4]arene with a free lower rim, like alkyl-substituted butyl calix[4]arene, forms stable submicron aggregates 150–200 nm in size, while the more lipophilic tetradecyl –substituted calix[4]arene forms micellar aggregates19 nm in size. Using UV-Vis spectroscopy, fluorimetry and CD, it was shown that amino-triazole calix[4]arenes bind to calf thymus DNA by classical intercalation. According to DLS and TEM data, all studied macrocycles cause significant DNA compaction, forming stable nanoparticles 50–20 nm in size. Among all studied calix[4]arenes the most lipophilic tetradecyl one proved to be the best for both binding and compaction of DNA.

## 1. Introduction

Gene delivery, the process of introduction foreign nucleic acids into host cells, is one of the main tools of evolution [1,2]. Artificial delivery of gene material is important from both scientific and practical biomedical points of view. The literature already contains examples of successful application of gene therapy using exogenous nucleic acids [3,4,5]. This technique has become widespread in the creation of vaccines based on messenger RNA [6]. By themselves, nucleic acids have a rather low ability to penetrate into cells—they can be subjected to degradation by enzymes. Therefore, one of the main problems in gene therapy is the search for safe and effective transfection agents that provide compaction and mask the negative charge of nucleic acids to ensure their effective delivery into the cell [7]. In addition to direct delivery methods, which are rarely effective [8], there are two main types of gene delivery technics: viral and non-viral. The latter are of particular interest [9] since they are not immunogenic unlike viruses. In addition, non-viral transfection agents allow the transfer of significant amounts of DNA [10]. The most commonly used non-viral vectors are cationic lipids, since they provide good levels of transfection, are structurally similar to cell membrane components, and are able to form complexes with negatively charged DNA (lipoplexes) due to their positive charge. Actually, the first systems for gene delivery were obtained just on the basis of cationic lipids [11] or cationic polymers [12]. Macrocyclic calixarene derivatives have great prospects for creating non-viral vehicles for delivery of nucleic acids [13,14,15,16,17]. According to Bohmer’s apt definition, calixarenes are macrocycles with ‘almost’ unlimited possibilities [18]. One striking demonstration of this definition is the amphiphilic calixarenes. They are unique systems with the ability to accommodate several polar fragments capable of efficient DNA binding at one core. Due to their three-dimensional structure, combining the properties of bola and gemini amphiphiles, amphiphilic derivatives are able to form highly organized lipid structures even at low concentrations. There are many successful examples in the literature of using cationic calixarenes for DNA binding, compaction and even storage. For this can be used calixarene-amines [19], multicalixarene-amines [20], calixdendrimer-amines [21], calixarenes bearing guanidinium [22,23,24] or arginine [25] groups as well as calixarene-ammonium derivatives [26,27,28]. We have previously demonstrated that thiacalixarene derivatives in the 1,3-alternate configuration carrying two [29] or four [30] diethylenetriamine fragments in combination with triazole fragments effectively bind and compact DNA.

Herein we present the synthesis of a new macrocycles based on the classical calix[4]arene in the cone configuration containing four diethylenetriamine fragments on the upper rim and free phenolic hydroxyl or alkyl-substituted groups on the lower rim, and the analysis of their interaction with model calf thymus DNA.

## 2. Results and Discussion

### 2.1. Synthesis

The introduction of azide groups into the macrocyclic platform is of interest because the resulting azides can be further modified with various moieties due to their unique reactivity [31]. One of the most powerful universal reactions, which allows to modify the macrocyclic platform, is the copper-catalyzed azide-alkyne cycloaddition reaction (CuAAC) [32,33]. Previously, we developed a method for the synthesis of di- and tetraarylazide derivatives based on the diazotization of available calix[4]arene di- and tetraamines, followed by substitution of the diazo group by an azide ion [34]. Carrying out this reaction with calixarene unsubstituted at the lower rim is also of great interest, since free phenolic hydroxyl groups can be post-modified by alkylation, acylation, etc. In addition, free phenolic hydroxyl groups are capable of hydrogen bonding and coordination of metal ions [35,36], which also makes it possible to design bifunctional structures.

**Scheme 1:**
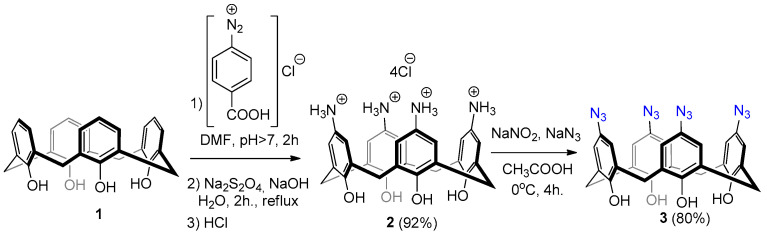
Synthetic approach for tetra-azide **3**.

For this (Scheme 1), calix[4]arene **1** was subjected to an azo coupling reaction with p-diazobenzoic acid chloride. The resulting azo-calixarene was reduced with sodium dithionite to obtain an amine hydrochloride **2** in almost quantitative yield. The diazotization reaction was carried out in acetic acid, stirring the mixture after adding sodium azide for 4 h, obtaining an azide **3** in high yield. The obtained azide turned out to be unstable in dry form; therefore, it was stored as a solution in methylene chloride. In the NMR ^1^H spectrum (Figure S1a) signals from bridging methylene protons appear as broadened singlets at 3.47 and 4.21 ppm. The signal of the aromatic protons appears as a singlet at 6.72 ppm. The signals of protons of hydroxyl groups at 9.95 ppm testify to the receipt of a macrocycle unsubstituted at the lower rim. The presence of azide fragments was clarified by intensive valent asymmetric bond vibrations at 2106 cm^−1^ in the IR spectra (Figure S1c). Composition of **3** was confirmed by high resolution electrospray ionization mass spectrometry (HR ESIMS). According to the obtained data (Figure S1d), in negative regime mono-anion [M-H]^1−^ with m/z 587.1657 (theoretical m/z = 587.1657) was found. Due to the known instability of aromatic azides [37], even with such a mild ionization method as electrospray, the formation of nitrene structures is also observed in the spectrum. For example, there is noticeable signal [M-H-4N_2_]^1−^ with m/z = 475.1413 (theoretical m/z = 475.1411). Crystals of the corresponding triethylammonium salt of the macrocycle **3**, suitable for X-ray diffraction analysis, were obtained by adding triethylamine to a solution of azide in dichloromethane.

**Figure 1:**
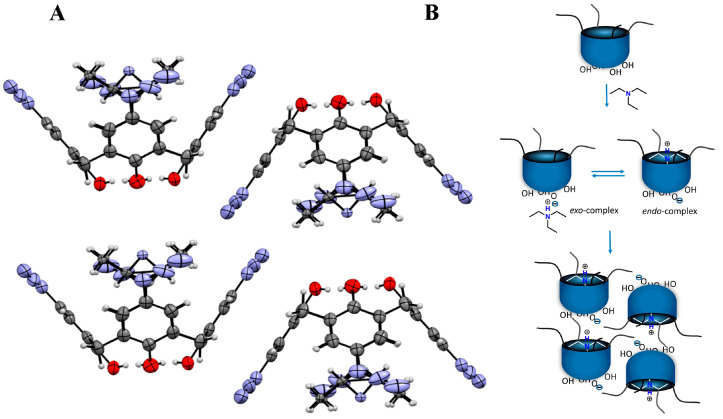
(**A**) ORTEP representation of **3** showing 20% probability thermal ellipsoids. C atoms—grey, O atoms—red, N atoms—blue. (**B**) Schematic representation of two different binding modes with triethylammonium cation.

**Figure 2:**
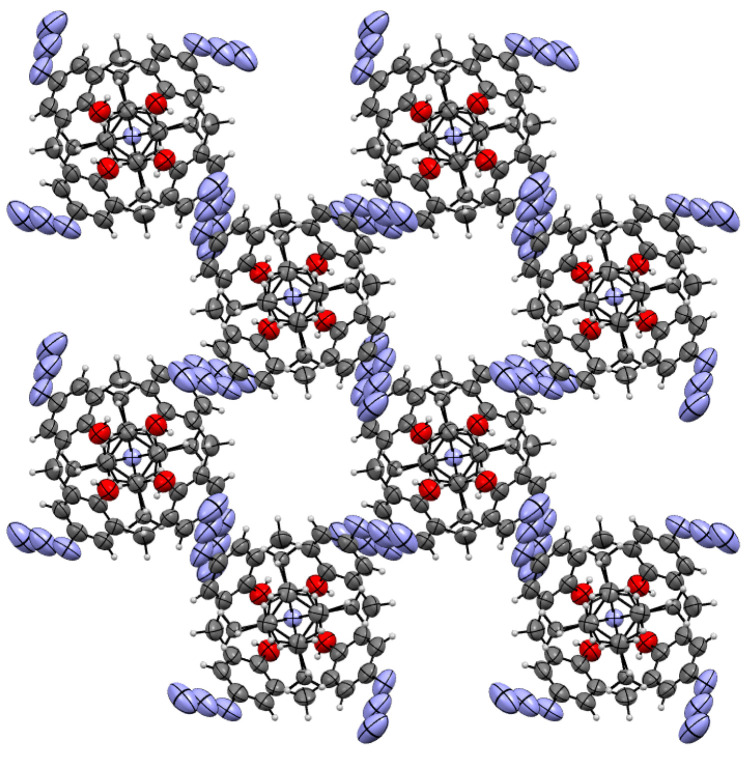
Crystal packing of **3** view along *c* axes.

In the resulting crystal (Figure 1A), the positions of the hydrogen atoms are not reliably specified. Therefore, the hydrogen atoms were inserted at the calculated positions and refined as riding atoms. However, taking into account the high acidity of phenolic hydroxyl groups in calixarenes [38] and their ability to form salts with amines [39] highly likely one hydroxyl hydrogen atom switch to triethylamine forming triethyl ammonium cation, located in the cavity of the one macrocycle, while interacting with the neighboring phenolate fragment of another macrocycle and thus represents a hybrid of the Gutsche’s [40] exo- and endo-types of the complexes (Figure 1B). A similar coordination was recently observed for the salt of *p-tert*-butyl calix[4]arene with the triethylmethylammonium cation [41]. The simplest cell of the crystal includes two calixarenes, whose aromatic fragments are parallel to each other (Figure 1A). The minimum distances between them, for example, between the atoms C008 and C006, are 3.379 Å, which corresponds to the classical π-π stacking [42]. It is noteworthy that the introduction of a triethylammonium fragment into the cavity does not lead to a cone configuration distortion—thus, the pairwise distance between the opposite carbon atoms of C008 is 8.391 Å, as well as the distance between opposite oxygen atoms O003 is 3.657 Å, which is obviously associated with the small size of the azide substituents and the ammonium salt itself, although for the above-mentioned *p-tert*-butylcalix[4]arene [41] such coordination causes a distortion of the cone configuration. Another reason for maintaining the undistorted cone configuration is the presence of a cyclic hydrogen bond on the lower rim, while in the tetra-O-butyl calix[4]arene tetraazide [34] a strong distortion of the configuration is observed—for example, the distance between opposite carbon atoms in this case is 10.051 (between C24 and C10) and 4.018 (between C3 and C17) due to the absence of a hydrogen bond. Additionally, the crystals of macrocycle **3** pack into an interesting cellular structure containing solvent molecules (CH_2_Cl_2_) in the cells (Figure 2).

**Scheme 2:**
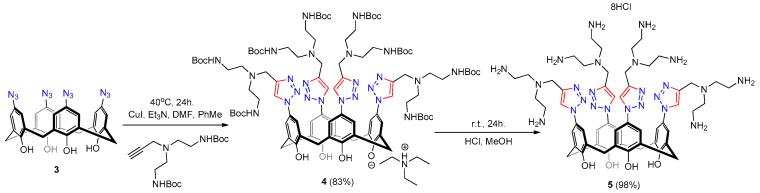
Synthetic approach for diethylenetriamine-containing derivative **5**.

The resulting tetraazide 3 was subjected to a CuAAC reaction with 3-bis[2-(*tert*-butoxycarbonylamino)ethyl]-propargylamine, which was carried out at 40 °C in a mixture of DMF-toluene for 24 h (Scheme 2) using a catalytic system of copper iodide-triethylamine. The product **4** was isolated in 83% yield as the mono-triethylammonium salt.

A broadened singlet corresponding to the triazole fragment proton signal appears at 7.85 ppm (Figure S2a). Broadened singlets corresponding to the signals of methylene protons at 3.83, 3.21, and 2.6 ppm as well as intense singlet at 1.39 ppm corresponding to the signal of *tert*-butyl group protons indicates the successful introduction of amine fragments into the structure. The intense signal of Boc C = O vibrations in FRIT spectra at 1697 cm^−1^ as well as the presence of [M-NEt_3_ + 2H]^2+^, [M-NEt_3_ + 3H]^3+^, [M-NEt_3_ + 4H]^4+^ ions in HRESI MS spectra in positive regime with m/z 978.0581, 652.3751 and 489.5331 (theoretical 978.0584, 652.3747 and 489.5328) unambiguously prove the composition of **4.** The Boc groups were removed by stirring a solution of compound **4** in methanol with hydrochloric acid. The obtained product was additionally purified by dialysis against disodium EDTA to remove copper residues. The signal of the *tert*-butyl groups protons disappears as a result of the Boc—protection removal (Figure S3a). It is noteworthy that the signal of the protons of the bridging methylene groups appears as a broad singlet at 4.60 ppm. A similar pattern is often observed in unsubstituted at the lower rim calixarene derivatives [43,44]. It’s associated with the low coalescence temperatures of unsubstituted calixarenes in polar solvents. According to Gutshe’s “broken chain” theory [45], a polar solvent, competitively participating in hydrogen bonding, breaks the cyclic hydrogen bond on the lower rim, leading to the conformational rotation of the macrocycle in solution. Taking into account the polyammonium nature of the compound, intense multiply charged ions are observed in the HRESI MS spectra in positive regime: [M + H]^+^, [M + 2H]^2+^, [M + 3H]^3+^, [M + 4H]^4+^ with m/z 1153.6864, 577.3460, 385.2320 and 289.1772 (theoretical 1153.6867, 577.3470, 385.2338, 289.1771).

**Scheme 3:**
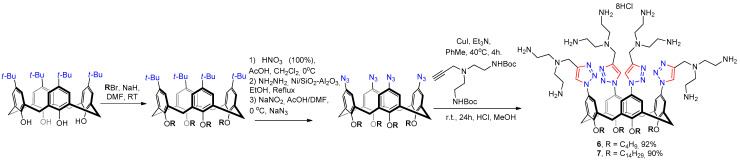
Synthesis of alkyl-substituted calixarenes **6**–**7**.

To evaluate the effect of the lipophilic substituent on the lower rim on the efficiency of binding, structuring, and compaction of model DNA, tetrabutyl- and tetradecyl-substituted polyamines **6** and **7** were compared against non-alkylated **5** (Scheme 3).

For this, calixarene **6** was synthesized using previously published methodology [34], including tetra-alkylation with butyl bromide, ipso-nitration, reduction using NH_2_NH_2_ in the presence of Ni on silica-alumina, diazotization with following substitution of diazo-groups by NaN_3_ and CuAAC reaction with 3-bis[2-(*tert*-butoxycarbonylamino)ethyl]-propargylamine with following Boc-deprotection. Calixarene **7** with four tetradecyl fragments was synthesized in the same manner using tetradecyl calixareneazide [46] instead of tetrabutyl one. The structure of calixarene **7** was well-proved using NMR, IR and HRESI MS. In the NMR ^1^H spectrum (Figure S4a) a strong broadening of all signals was found even in DMSO-d6, which is typical for amphiphilic calixarene derivatives due to strong aggregation. The signals of the triazole and aromatic protons appear as singlets at 8.63 and 7.34 ppm, respectively. Signals of methylene protons appear as broad singlet at 3.79 and two broad multiplets at 3.03–2.95 and 2.68–2.60 ppm. In the HRESI MS spectrum (Figure S4d), intense multiply charged ions are observed in positive regime: [M + H]^+^, [M + 2H]^2+^, [M + 3H]^3+^, [M + 4H]^4+^ with m/z 1939.5665, 970.2862, 647.1931 and 485.6463 (theoretical 1939.5665, 970.2869, 647.1937, 485.6471).

### 2.2. Aggregation Behavior

**Scheme 4:**
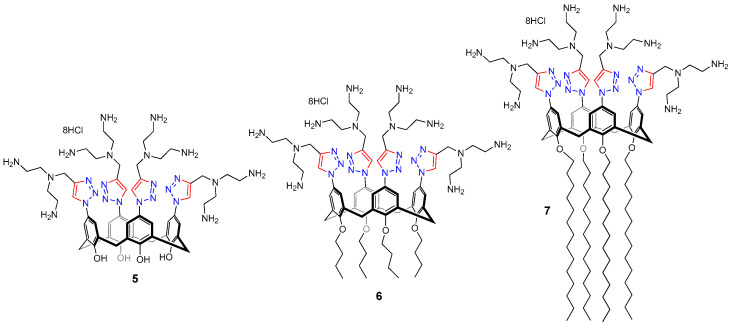
Structure of the investigated polyammonium calixarenes **5**–**7**.

The critical aggregation concentration (CAC) values of macrocycles **6**–**7** (Scheme 4) were determined using fluorophore pyrene probe. The CAC values can be determined by a plot of the pyrene emission intensity as a function of the macrocycle concentration (Figure S5, Table 1) [47]. The typical behavior for calix[4]arenes containing positively charged headgroups on the upper and hydrophobic moieties on the lower rims in aggregates formation in the aqueous solutions was observed [48]. Thus, the CAC values for calixarenes **6** and **7** are relatively close and one order of magnitude lower than the corresponding values for previously reported isostructural triazole calixarenes containing carboxylate polar groups [46] and are comparable with the values obtained for amphiphilic calix[4]arenes with polar hydroxyalkylammonium groups in the TRIS solution [49]. As for macrocycle **5**, it was impossible to measure it’s CAC values using a hydrophobic probe due to the absence of a typical amphiphilic structure of the latter. The aggregate formation of macrocycles in aqueous solution was studied by dynamic light scattering (DLS) method. Surprisingly, it was found that macrocycle **5** forms stable aggregates with the average diameter of 220 nm and low polydispersity index (PDI). As for the amphiphilic macrocycles **6** and **7**, a bimodal distribution of particle with the sizes around 200 nm and about 10–15 nm was observed in both cases. It is noteworthy that lipophilicity significantly affects the distribution of aggregates of different sizes: while butyl derivative **6** forms predominantly large vesicle-like aggregates (73% by intensity) with a size of 146 nm, more lipophilic **7** is represented mainly in small micelles (74% by intensity) with a size of 16 nm (Figure S6). An increase of the macrocycle alkyl substituents length leads to a more efficient hydrophobic interaction and the formation of denser compact particles as it was observed earlier [46].

The size of the system containing macrocycle **5** does not change upon transition to the acidic pH region. An acidic pH results in only a slight increase of the surface potential due to greater protonation of the amino groups. Upon transition to the alkaline pH region, the formation of micron-sized (1.6 μm) unstable aggregates and recharging of the system are observed. Thus, the structuring of the system in the range of neutral and acidic pH results from a complex system of hydrogen bonds between protonated ammonium fragments with phenolic hydroxyl groups and water [50]. Upon transition to the region of alkaline pH, ammonium groups and partially phenolic hydroxyl groups are deprotonated [51], which results in a significant destabilization of the system.

### 2.3. CT DNA Binding Abilities

#### 2.3.1. UV Absorption Spectroscopic Study

**Figure 3:**
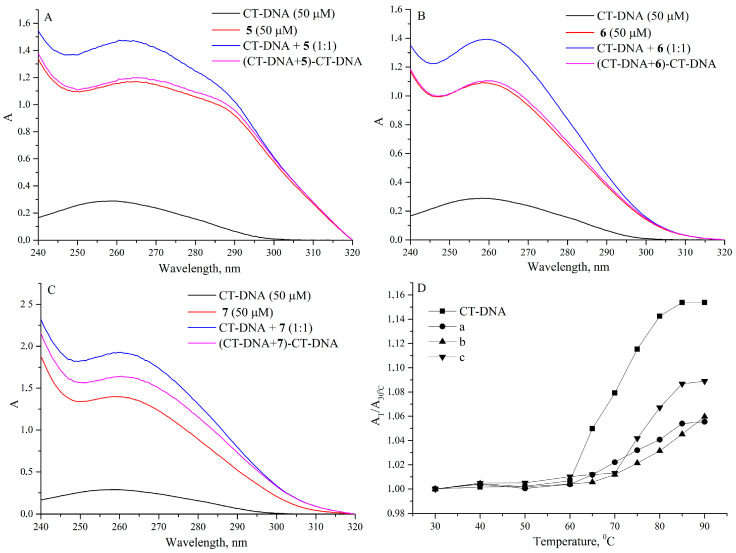
The UV absorption spectra for CT-DNA (50 μM), CT-DNA + macrocycle complex (1:1) (**A**) **5**, (**B**) **6**, (**C**) **7**, and the difference spectrum. (**D**): melting curve for CT-DNA alone (50 μM) and in the presence of a—**5** (50 μM), b—**6** (50 μM), c—**7** (50 μM).

The common method for quantification of intermolecular interaction DNA with various ligands is absorption spectroscopy. Typical UV–vis absorption spectrum of calf thymus-DNA (CT-DNA) with absorption maximum at 260 nm reveals a broadband in the UV region (Figure 3). The corresponding maximum is associated with electronic transitions of chromophore groups in pyrimidine (cytosine and thymine) and purine (adenine and guanine) moieties. Conformational changes in the DNA helix upon interaction with ligands can be detected through red/blue shift, hyperchromic/hypochromic effects in UV-vis spectra [21]. The UV-vis spectra for free macrocycles **5**–**7** represents a characteristic π-π* transition absorption peaks at 265 nm, 258 nm and 258 nm, respectively. The redshift of macrocycles **5**–**7** after addition to CT-DNA was found as 3 nm, 5 nm and 5 nm, respectively. The absorption peak of CT-DNA at 260 nm also underwent a hyperchromic effect after the addition of macrocycles. The hyperchromic effect and the shift of the absorption maximum indicate the formation of complexes between CT-DNA and macrocycles.

The melting temperature of DNA (T_m_) is defined as the temperature at which half of the DNA double-helical structure unfolds into two individual strands. Changes in the T_m_ of DNA provide strong evidence of an interaction leading to stabilization or destabilization of the helix. DNA melting in the presence of a binding partner can also be used to distinguish between intercalative and external binding methods [52]. The T_m_ values were determined by the transition midpoint from the melting curve obtained in the UV-vis temperature experiment (Figure 2D). For free CT-DNA (50 μM), T_m_ was found as 72.5 °C, while in the presence of macrocycles **5**–**7** (50 μM) it was found to be 75.1 °C, 77.5 °C and 84.2, respectively. Since classical intercalation results in higher T_m_ values compared to groove bonding or overlaying, the results imply thermal stabilization of the polynucleotide by the macrocycle intercalation.

#### 2.3.2. Fluorescence Spectroscopic Studies

**Figure 4:**
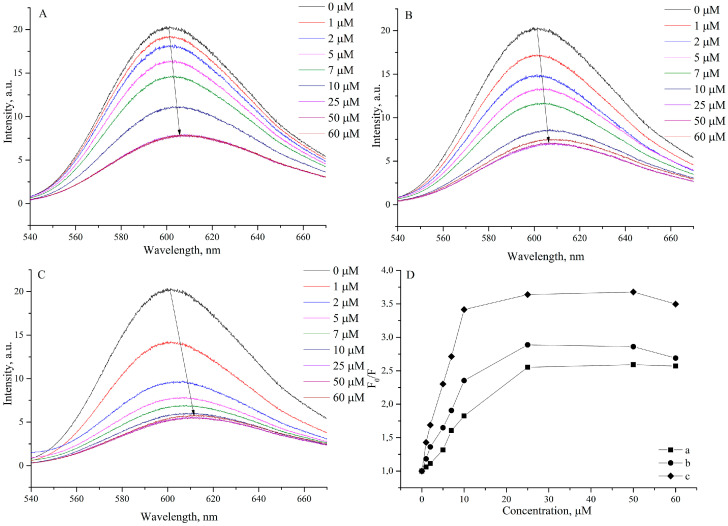
Emission spectra of the EthBr–CT-DNA system in the absence and presence of various concentrations of the macrocycles (**A**)—**5**, (**B**)—**6**, (**C**)—**7** in 10 mM Tris/50 mM NaCl buffer solution with pH 7.4. [CT DNA] = 50 μM, [EthBr] = 10 μM, λ_ex_ = 520 nm. (**D**)—plot of F_0_/F versus [macrocycle], a—**5**, b—**6,** c—**7**.

Competitive binding of ethidium bromide (EthBr) and **5**–**7** to CT-DNA was used to assess interactions between DNA and macrocycles using fluorescent emission spectroscopy. EthBr intercalates into DNA by base-pair stacking, which leads to a strong increase in the fluorescence intensity of EthBr [53]. During competitive binding EthBr is displaced from DNA, which leads to a fluorescence quenching. Consequently, the degree of EthBr quenching can be used to determine the extent of binding between the macrocycle and DNA. On Figure 4 shows the fluorescence spectra of DNA–EtBr complexes with different concentrations of macrocyles.

An increase in the concentration of macrocycles leads to quenching of the DNA–EthBr complex fluorescence. Such a change in the fluorescence intensity indicates that macrocycles can replace EthBr from the DNA–EthBr system. The classical Stern–Volmer equation can be used to assess qualitatively quenching extent (Equation (1)) [54], where [*Q*] is the concentration of macrocycle (quencher), *F_0_* and *F* are the fluorescence intensities in the absence and presence of the macrocycle, respectively, K_q_ is the bimolecular quenching constant and *τ_0_* is the lifetime of the fluorophore in the absence of quencher.

(1) $$F_{0}/F=1+K_{q}\tau_{0}\left[Q\right]=1+K_{SV}\left[Q\right]$$

Since fluorescence lifetime of EthBr is typically near 10^−8^ s [55], the bimolecular quenching constant (*K_q_*) can be simply calculated from *K_sv_* = *K_q_* × 10^−8^ s. The *K_sv_* can be calculated from the ratio of the slope of the titration curve to the point of intersection. The fluorescence quenching curve of the DNA-bound EtBr by macrocycles (Figure 3D) is in good agreement with the linear curve of the Stern–Volmer equation at low concentrations (1–10 μM), thus suggesting that only one mode for causing fluorescence quenching can be assumed. At concentrations of macrocycles above 10 µmol, the emission of EthBr reaches a plateau. Taking into account the concentration of EthBr (10 µmol), in this region already most of the EthBr turns out to be substituted by the macrocycle. The values of *K_sv_* are given in Table 2. According to Equation (1), *K_q_* is higher than the limiting diffusion rate constant (2.0 × 10^10^ M^−1^ s^−1^) for a biomacromolecule, which indicates the existence of a static quenching mechanism [56].

For static quenching, the relationship between fluorescence intensity and the quencher concentrations can be expressed by the following double log regression equation [57] (Equation (2)):

(2) $$\log\frac{F_{0}-F}{F}=\log K_{a}+n\log C$$

where *K_a_* is the binding constant and *n* is the number of binding sites.

K_a_ and *n* of **5**–**7** with DNA were calculated from double logarithm regression curves (Table 2, Figure S7). The value of the *K_a_* constant shows that the length of the alkyl substituent in macrocycles is critical for effective DNA binding. Thus, if for macrocycle **5** and **6** the values of *K_a_* are close, then in the case of tetradecyl **7** *K_a_* increases by an order of magnitude. The binding sites *n* of **5**–**7** with DNA were calculated as 0.74, 0.83 and 1.17, respectively. The results are in good agreement with the data obtained in a series of conventional cationic single-chain surfactants [58], where the authors attribute a significant increase in the efficiency of interaction with DNA to an increase in lipophilicity and a decrease in the CAC value.

#### 2.3.3. Circular Dichroism Spectral Studies

**Figure 5:**
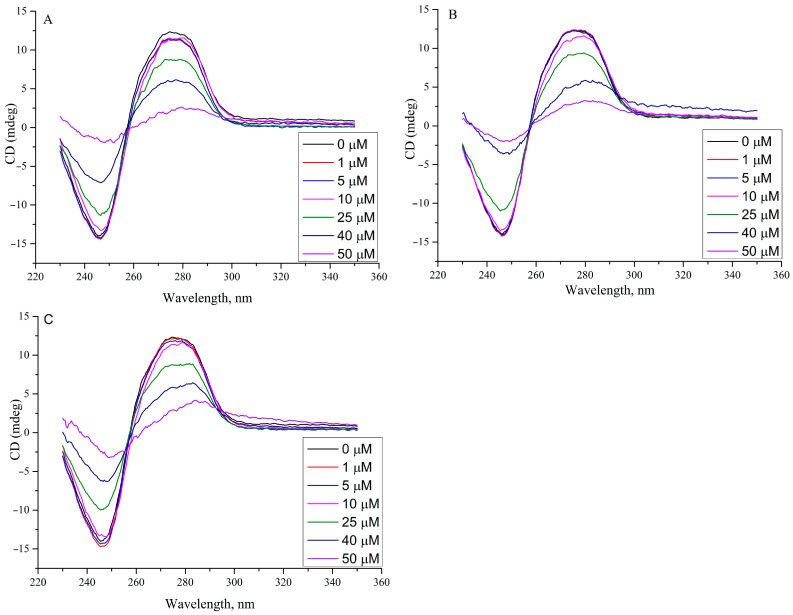
Circular dichroism (CD) spectra of CT-DNA (50μM) in the absence a and presence of different amounts of the macrocycles (**A**)—**5**, (**B**)—**6**, (**C**)—**7**.

The circular dichroism (CD) spectra of double-stranded CT-DNA exhibit a positive CD band at 275 nm for the nucleobase stacking and a negative band at 246 nm for the helix B conformation (Figure 5) [59].

Upon the addition of the macrocycle to the CT-DNA solution, the intensity of both positive and negative CD bands decreases and shifts to the red region to 286 and 250 nm, respectively. The observed redshift with a decrease in intensity during complexation can be attributed to a structural transition from the B- to C form, accompanied by the DNA compaction process, which was previously shown for cationic surfactants [60,61].

#### 2.3.4. Particle Size and Zeta Potential Measurements

**Figure 6:**
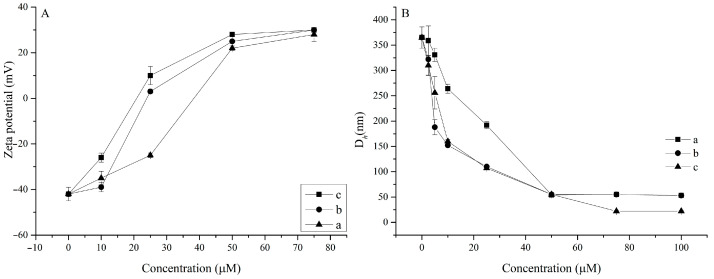
Zeta potential (**A**) and particle size (**B**) of CT-DNA in the presence of different amounts of macrocycles **5** a, **6** b and **7** c. [DNA] = 50 μM.

The above spectroscopic studies, including UV, fluorescence and CD, showed that macrocycles **5**–**7** can effectively interact with CT DNA. Previously, it was shown that amphiphilic macrocycles with diethylenetriamine groups are capable of compacting CT DNA into small globules [29,30]. The particle size and zeta potential of the CT DNA-macrocycle complexes with different concentrations were studied by the DLS method (Figure 6).

According to the data obtained, the concentration of macrocycles has a significant effect on both zeta potential and the particle size. The zeta potential and average hydrodynamic diameter of CT-DNA without macrocycles were found as −32 mV and 358 nm, respectively. When macrocycles were added at a concentration of 25 μM, the surface potential was recharged, reaching a plateau with values of about 30 mV. The negative charges of DNA can be gradually neutralized by cationic amphiphilic macrocycles. Thus, with an increase in its concentration, cationic amphiphilic macrocycles first bind CT DNA through electrostatic interaction, and then assemble into micelles or quasi-micelles (lipoplexes) with a concentrated positive charge. The zeta potential plot clearly shows that the more lipophilic macrocycle **7** causes surface recharge at lower concentration, which is directly related to its lower CAC. Despite the complete absence of alkyl substituents, macrocycle **5** also causes surface recharging at higher concentrations. As for the change in CT DNA size in the presence of macrocycles, DNA compaction to particles of 54 nm in size is observed at an equimolar concentration of macrocycles (50 μM). A further increase in concentration continues compaction only in the case of the more lipophilic macrocycle **7** to give fine particles of 22 nm in size (Figure S8). The size of DNA complex has been considered one of the most important limiting factors for nonviral gene vectors. The requirement for strict control of the complex size is largely due to the size limitation of 150 nm for endocytosis and escape from the blood vessel [62]. Thus, the DNA complexes with the studied macrocycles meet the size requirements.

#### 2.3.5. Transmission Electron Microscopy (TEM)

**Figure 7:**
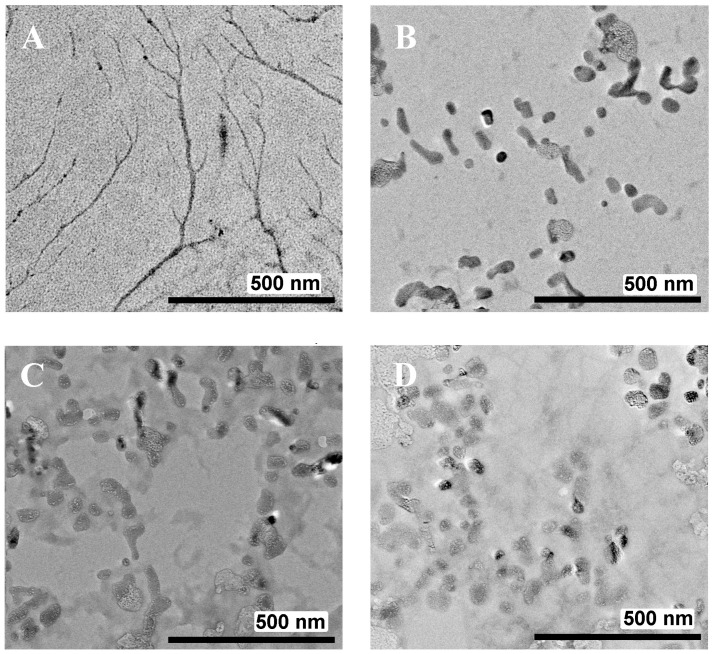
TEM images of CT-DNA (**A**), CT-DNA with **5** (**B**), CT-DNA with **6** (**C**), CT-DNA with **7** (**D**).

The surface morphology of the macrocycle/CT-DNA systems was observed using TEM. The structures of free CT-DNA and macrocycles/CT-DNA combinations are shown in Figure 7.

**Scheme 5:**
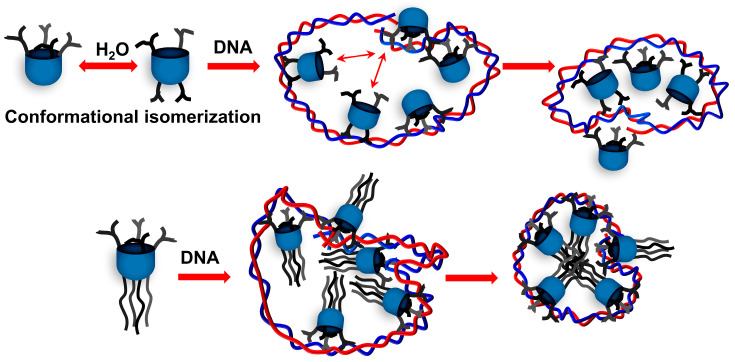
DNA binding mode for calix[4]arenes with/without lipophilic substituents on the lower rim.

Images of free CT-DNA show a typical fibril-like structure [63]. The formation of globular structures with sizes in the range of 50–20 nanometers is observed in the presence of macrocycles in binary systems. The data obtained are in full agreement with other experiments, including DLS. Notably, macrocycle **5** also successfully condenses DNA. Taking into account it’s conformational mobility, demonstrated in the synthesis section, it is most likely that calixarene **5** is able to adopt bitopic conformations (for example, 1,3-alternate or partial cone), which leads to efficient binding of different DNA regions (Scheme 5).

## 3. Materials and Methods

### 3.1. Characterisation Methods

^1^H and ^13^C NMR spectra as well as 2D ^1^H-^1^H NOESY were recorded on Bruker Avance 400 Nanobay (Bruker Corporation, Billerica, MA, USA) with signals from residual protons of CDCl_3,_ D_2_O or DMSO-d_6_ as internal standard.

The melting points were measured using the Optimelt MPA100 melting point apparatus (Stanford Research Systems, Sunnyvale, CA, USA).

IR spectra in KBr pellets were recorded on a Bruker Vector-22 spectrometer (Bruker Corporation, Billerica, MA, USA).

High-resolution mass spectra with electrospray ionization (HRESI MS) were obtained on an Agilent iFunnel 6550 Q-TOF LC/MS (Agilent Technologies, Santa Clara, CA, USA) in positive or negative mode. Carrier gas-nitrogen, temperature 300 °C, carrier flow rate 12 l × min^−1^, nebulizer pressure 275 kPa, funnel voltage 3500 V, capillary voltage 500 V, total ion current recording mode, 100–3000 m/z mass range, scanning speed 7 spectra × s^−1^.

### 3.2. Reagents

Chemicals were purchased from commercial suppliers and used as received. Solvents were purified according to standard methods. 3-Bis[2-(*tert*-butoxycarbonylamino)ethyl]propargylamine, 25,26,27,28-tetrahydroxycalix[4]arene, 5,11,17,23-tetraazo-25,26,27,28-tetrahydroxycalix[4]arene, 5,11,17,23-tetraamino-25,26,27,28-tetrahydroxycalix[4]arene [64,65,66] and 5,11,17,23-tetra(4-((bis(2-(amino)ethyl) amino)methyl)-1H-1,2,3-triazol-1-yl))-25,26,27,28-tetrabutylcalix[4]arene octahydrochloride (**6**) [34] were prepared following literature procedures.

Synthesis of 5,11,17,23-tetraazide-25,26,27,28-tetrahydroxycalix[4]arene (**3**):

Compound **2** (1 mmol) was dissolved in H_2_O/glacial acetic acid (3:10 *v*/*v*, 32 mL). Then, the mixture was cooled to 0 °C and 0.28 g of sodium nitrite (4 mmol), dissolved in cold water, (6.5 mL) was added dropwise. The mixture was stirred for 30 min and 0.52 g of sodium azide (8 mmol) in 36 mL of water was added. Gas evolution was observed during this stage. The solution was stirred for another 4 h, then, 100 mL of cold water was added. The product was extracted with CHCl_3_ (2 × 50 mL) and the combined organic phase was dried over Na_2_SO_4_. The mixture was filtered; the filtrate was dried on a rotary evaporator. As a result, a red-brown precipitate was obtained. Yield 0.47 g (80%); mp 71 °C (decomp.).

^1^H NMR (400 MHz, CDCl_3_*,* 25 °C) δ 9.94 (s, 4H, Ar-OH), 6.71 (s, 8H, ArH), 4.21 (brs, 4H, Ar-CH_2_-Ar), 3.47 (brs, 4H, Ar-CH_2_-Ar).

^13^C NMR (101 MHz, CDCl_3,_ 25 °C) δ 146.08, 134.52, 129.19, 119.57, 31.83

IR (KBr) ν _max_ cm^−1^ (triethylammonnium salt of 4): (CH_2_), 2854 (CH_2_) 2122 (N_3_), 1644 (C-C (Ar)), 1493 (CH_2_)

HRESI MS (m/z) [M-H]^1−^: calcd. for [C_28_H_19_N_12_O_4_]^1-^: 587.1657, found: 587.1657

Crystal Data for C_34_H_35_N_13_O_4_ (*M* = 689.75 g/mol): tetragonal, space group P4/*n* (no. 85), *a* = 14.5497(4) Å, *c* = 9.7360(4) Å, *V* = 2061.05(13) Å^3^, *Z* = 2, *T* = 100.00(10) K, μ(Cu Kα) = 0.635 mm^−1^, *Dcalc* = 1.111 g/cm^3^, 58,939 reflections measured (8.594° ≤ 2Θ ≤ 154.314°), 2134 unique (*R*_int_ = 0.1231, R_sigma_ = 0.0276) which were used in all calculations. The final *R*_1_ was 0.1806 (I > 2σ(I)) and *wR*_2_ was 0.5026 (all data). CCDC number 2215732.

Synthesis of 5,11,17,23-tetra(4-((bis(2-((tert-butoxycarbonyl)amino)ethyl)amino)methyl)-1H-1,2,3-triazol-1-yl))-25,26,27,28-tetrahydroxycalix[4]arene triethylammonium salt (**4**):

1 mmol of compound **3**, 1.36 g (4 mmol) 3-bis[2-(*tert*-butoxycarbonylamino)ethyl]propargylamine, 0.012 g (0.06 mmol) CuI and 3.0 g of triethylamine (30 mmol) were dissolved in 30 mL of dry toluene/DMF solution (5:1) and N_2_ was bubbled through the solution for 10 min. The reaction mixture was stirred at 40 °C for 24 h. The solvent was evaporated in vacuo. The obtained residue was then dissolved in CHCl_3_ (80 mL) and washed with water (2 × 100 mL). The organic layer was dried over Na_2_SO_4_ and the solvent was evaporated in vacuo. The crude product was precipitated in methanol/diethyl ether to give product **4** as brown powder. Yield 2.11 g (83%); mp 191 °C (decomp.).

^1^H NMR (400 MHz, CDCl_3_*,* 25 °C) δ 7.85 (s, 4H, TrzH), 7.31 (s, 8H, ArH), 5.15 (brs, 8H, NH), 4.64 (brs, 4H, Ar-CH_2_-Ar), 3.83 (brs, 8H, Trz-CH_2_-N-), 3.48 (brs, 4H, Ar-CH_2_-Ar), 3.21 (brs, 16H, N-CH_2_-), 2.60 (brs, 16H, -CH_2_-N), 2.38 (brs, 6H, Et_3_N), 1.89 (s, 72H, t-Bu), 0.42 (brs, 9H, Et_3_N).

^13^C NMR (101 MHz, CDCl_3,_ 25 °C) δ 179.34, 156.41, 131.47, 128.19, 122.27 121.49, 79.37, 73.46, 53.44, 52.86, 48.28, 46.24, 38.29, 33.02, 28.53.

IR (KBr) ν _max_ cm^−1^: 3336 (OH), 2976 (CH_2_), 2932 (CH_2_), 1696 (C = O), 1494 (CH_2_), 1392 (C(CH_3_)_3_), 1366 (C(CH_3_)_3_), 1250 (C-N), 1169 (C-O).

HRESI MS (m/z) [M-NEt_3_+2H]^2+^, [M-NEt_3_+3H]^3+^, [M-NEt_3_+4H]^4+^: calcd. for [C_96_H_146_N_24_O_20_]^2+^: 978.0584, found: 978.0581; calcd. for [C_96_H_147_N_24_O_20_]^3+^: 652.3747 m/z, found: 652.3751; calcd. for [C_96_H_148_N_24_O_20_]^4+^: 489.5328 m/z, found: 489.5331.

Synthesis of 5,11,17,23-tetra(4-((bis(2-(amino)ethyl) amino)methyl)-1H-1,2,3-triazol-1-yl))-25,26,27,28-tetrahydroxycalix[4]arene octahydrochloride (**5**)

1.0 mmol of compound **4** was dissolved in 100 mL of methanol, and then 8 mL of 37% hydrochloric acid was added dropwise. The reaction mixture was stirred at room temperature for 24 h. The residue was filtered and filtrate was evaporated in *vacuo*. The crude product was precipitated by 20 mL of methanol to give product **5** as beige powder. Then, product was dissolved in 5 mL of deionized water and was dialyzed against 0.01 M disodium EDTA and then against pure water. After evaporation a beige powder was isolated. Yield 1.41g (98%), mp 251 °C (decomp.).

^1^H NMR (400 MHz, D_2_O, 25 °C) δ 8.78 (s, 4H, TrzH), 7.82 (s, 8H, ArH), 4.60 (brs, 8H, Ar-CH_2_-Ar), 4.12 (s, 8H, Trz-CH_2_-N), 3.63–3.48 (m, 32H, -N-CH_2_-)

NMR (101 MHz, DMSO d6-methanol-d4 10:1, 25 °C) δ 152.05, 141.53, 130.64, 130.27, 124.83, 122.10, 50.57, 46.83, 36.47, 31.55.

IR (KBr) ν _max_ cm^−1^: 3394 (OH), 2960 (Ar+NH), 1603 (NH), 1492 (CH_2_), 1459 (CH_2_).

HRESI MS (m/z) [M+H]^+^, [M+2H]^2+^, [M+3H]^3+^, [M+4H]^4+^: calcd. for [C_56_H_81_N_24_O_4_]^+^: 1153.6867, found: 1153.6864; calcd. for [C_56_H_82_N_24_O_4_]^2+^: 577.3470, found: 577,3460; calcd. for [C_56_H_83_N_24_O_4_]^3+^: 385.2338, found: 385,2320, calcd. for [C_56_H_84_N_24_O_4_]^4+^: 289.1771, found: 289,1772.

Synthesis of 5,11,17,23-tetra(4-((bis(2-(amino)ethyl) amino)methyl)-1H-1,2,3-triazol-1-yl))-25,26,27,28-tetratetradecylxycalix[4]arene octahydrochloride (**7**)

Synthesis was performed using the same methodology as previously published for **6** [23] using tetradecyl calixareneazide [32].

^1^H NMR (400 MHz, DMSO-d_6_*,* 25 °C) δ 8.63 (s, 4H, TrzH), 8.01 (brs, 16H, NH), 7.34 (s, 8H, ArH), 4.45 (d, 4H, Ar-CH_2_-Ar), 3.99–3.91 (m, 8H, O-CH_2_), 3.79 (brs, 8H, Trz-CH_2_-N), 3.03–2.95 (m, 16H, -N-CH_2_-), 2.68–2.60 (m, 16H, -N-CH_2_-), 1.99–1.87 (m, 20H, -CH_2_-), 1.29–1.19 (m, 84H, -CH_2_-), 0.87–0.81 (m, 12H, -CH_3_).

^13^C NMR (101 MHz, DMSO-d_6_, 25 °C) δ 179.52, 155.98, 135.57, 131.30, 122.86, 119.94, 75.33, 49.92, 46.18, 39.52, 36.16, 31.34, 29.91, 29.67, 29.50, 29.40, 29.34, 29.28, 29.17, 28.81, 26.06, 22.09, 13.83.

HRESI MS (m/z) [M+H]^+^, [M+2H]^2+^, [M+3H]^3+^, [M+4H]^4+^: calcd. for [C_112_H_193_N_24_O_4_]^+^: 1939.5665, found: 1939.5670; calcd. for [C_112_H_194_N_24_O_4_]^2+^: 970.2869, found: 970.2862; calcd. for [C_112_H_195_N_24_O_4_]^3+^: 647.1937, found: 647.1931, calcd. for [C_112_H_196_N_24_O_4_]^4+^: 485.6471, found: 485.6463.

### 3.3. Sample Preparation

The stock solutions of macrocycle (1 mM) and EtBr (1 mM) were prepared in distilled water. A few threads of CT-DNA were dissolved in 10 mM Tris-HCl buffer (pH 7.4) and kept at 4 °C for 24 h. The solution was timely stirred to form a homogenous solution. The concentration of CT-DNA per nucleotide was determined using a mean extinction coefficient of 6600 M^−1^ cm^−1^ at A_260 nm_ [X]. The ratio of absorbance (A_260 nm_/A_280 nm_) was found to be 1.8, ensuring the purity of CT-DNA. The working solutions were made by dilution method as per requirement. All experiments were conducted in 10 mM Tris-HCl buffer (pH 7.4).

### 3.4. Instrumentations

Shimadzu UV-visible spectrometer UV-2600 (Shimadzu Corporation, Kyoto, Japan) was used to perform the UV-visible absorption studies using 1.0 cm quartz cuvettes. Fluorolog FL-221 spectrofluorimeter (HORIBA Jobin Yvon, Kyoto, Japan) was employed to perform fluorescence spectroscopic experiments using 1.0 cm quartz cuvettes. The CD spectra were recorded on a J-1500 CD (Jasco, Tokyo, Japan) spectrophotometer equipped with a Peltier temperature controller maintained at 25 °C, using a quartz cuvette of path length 1.0 cm.

### 3.5. Dynamic and Electrophoretic Light Scattering Study

DLS and ELS experiments were carried out on Zetasizer Nano ZS instrument (Malvern Panalytical, Worcestershire, UK) with 4 mW 633 nm He–Ne laser light source and the light scattering angle of 173°. The data were treated with DTS software (Dispersion Technology Software 5.00). The solutions were filtered through 0.8 µm filter before the measurements to remove dust. The experiments were carried out in the disposable plastic cells DTS 0012 (size) or in the disposable folded capillary cells DTS 1070 (zeta potential) (Sigma-Aldrich, USA) at 298 K with at least three experiments for each system. Statistical data treatment was done using t-Student coefficient and the particle size determination error was <2%.

### 3.6. UV Absorption Spectroscopic Study

The difference spectra between the macrocycles/CT-DNA complex and CT-DNA were obtained in 1:1 M ratio (50 μM). The spectra were recorded between 240 and 320 nm, using a quartz cuvette of path length 1 cm.

The melting curve was plotted by measuring the absorbance of CT-DNA or the mixture of CT-DNA (50 μM) and the macrocycles (50 μM) at 260 nm at various temperatures using a UV–Vis spectrophotometer. The melting point, T_m_, was obtained as the transition midpoint from the melting curve.

### 3.7. Steady-State Fluorescence Study

CAC values were measured using pyrene fluorescent probe and calculated from the dependence of the intensity ratio of the first (373 nm) and third (384 nm) bands in the emission spectrum of pyrene vs. calixarene concentration. Fluorescence experiments with pyrene were performed in 10.0 mm quartz cuvettes and recorded in the range of 340 to 430 nm and excitation wavelength 335 nm with 2.5 nm slit. All studies were conducted in buffered aqueous solution (TRIS buffer, pH 7.4) at 298 K. For the preparation of DNA/EtBr mixture, 10 μM EtBr and 50 μM DNA were mixed and incubated at 25 °C for 2 h. Then, the same volume of macrocycles solutions with different concentrations were added to the above prepared DNA/EtBr mixture solution and left for 2 h. Fluorescence emission was measured in the wavelength range of 540–670 nm with excitation wavelength at 480 nm and 2.5 nm slit.

### 3.8. Circular Dichroism (CD) Study

CD experiments were performed to investigate the conformation change of DNA. All the CD spectra of different samples were recorded in the wavelength range of 220 to 320 nm at 25 °C by taking points at 0.5 nm. Each spectrum was the average of three runs at 25 °C and a five minute equilibration before each scan.

### 3.9. Transition Electron Microscopy (TEM) Study

TEM was performed on Hitachi HT7700 ExaLens (Hitachi High-Tech Corporation, Tokyo, Japan) in Interdisciplinary Center for Analytical Microscopy of Kazan Federal University. The images were acquired at an accelerating voltage of 100 kV. Samples were ultrasonicated in water for 10 min, dispersed on 200 mesh copper grids with continuous formvar support films and then dried during 3 h.

### 3.10. X-ray Study

Data set for single crystal **3** was collected on a Rigaku XtaLab Synergy S instrument (Rigaku Corporation, Tokyo, Japan) with a HyPix detector and a PhotonJet microfocus X-ray tube using Cu Kα (1.54184 Å) radiation at low temperature. Images were indexed and integrated using the CrysAlisPro data reduction package. Data were corrected for systematic errors and absorption using the ABSPACK module: numerical absorption correction based on Gaussian integration over a multifaceted crystal model and empirical absorption correction based on spherical harmonics according to the point group symmetry using equivalent reflections. The GRAL module was used for analysis of systematic absences and space group determination. The structure was solved by direct methods using SHELXT [67] and refined by the full-matrix least-squares on F^2^ using SHELXL [68]. Non-hydrogen atoms were refined anisotropically. The hydrogen atoms were inserted at the calculated positions and refined as riding atoms. A solvent mask was calculated and 166 electrons were found in a volume of 466 Å^3^ in 1 void per unit cell. This is consistent with the presence of 4[CH_2_Cl_2_] per unit cell which account for 168 electrons per unit cell. The figures were generated using Mercury 4.1 [69] program. Crystals were obtained by slow evaporation method.

## 4. Conclusions

A new precursor of click chemistry, calix [4]arene tetraazide with free lower rim, has been synthesized. It was further used in the synthesis of water-soluble calixarene triazole containing 12 aminogroups. In addition, to assess the role of lipophilicity and amphiphilic architecture in the binding of the model CT-DNA, amphiphilic calixarenes containing lipophilic butyl or tetradecyl fragments on the lower rim and the same amino-triazole groups on the upper rim were also synthesized. Aggregation in a series of isostructural amino-triazole macrocycles of different lipophilicity was studied. It was found that calixarene with a free lower rim like alkyl-substituted butyl calixarene, forms stable submicron aggregates 150–200 nm in size, while the more lipophilic tetradecyl one forms micellar aggregates 19 nm in size. The CAC values for calixarenes with butyl or tetradecyl fragments turned out to be very close and amount to 5 and 3.4 μM, respectively. According to UV absorption spectroscopic study of CT-DNA–calixarene systems including CT-DNA melting temperature measurements and fluorometry with ethidium bromide, it was shown that amino-triazole calixarenes bind CT-DNA by classical intercalation, and the most lipophilic tetradecyl calixarene turned out to be the most effective: the value of K_a_ constant turned out to be an order of magnitude higher than for unsubstituted and butyl-substituted calixarenes. According to CD measurements, upon complexation with calixarenes, CT-DNA undergoes structure transition from the B- to the C form. All studied macrocycles cause significant compaction of CT-DNA, forming stable 54 nm nanoparticles in the case of unsubstituted and butyl-substituted macrocycles and 22 nm nanoparticles in the case of tetradecyl calixarene, which are currently the most compact DNA-calixarene lipoplexes [13,16]. Resuming, the amphiphilic tetradecyl calixarene has the highest affinity for CT-DNA in the series studied. Due to its conformational mobility, calixarene amine with free lower rim also effectively compacts CT-DNA, adopting a convenient conformation. The results obtained have great prospects for the practical development of nucleic acid delivery or storage systems.

## Figures and Tables

**Table 1:** DLS, ELS and CAC data for **5–7** solutions ^1^.

System	CAC, μM	D Average, nm	d1, nm	d2, nm	PDI	ζ, mV
**5**	-	221 ± 8	-	-	0.256 ± 0.017	+42 ± 6
**5 ^2^**	-	167 ± 32	-	-	0.331 ± 0.042	+46 ± 2
**5 ^3^**	-	1679 ± 83	2548 ± 79 (78%)	443 ± 42 (22%)	0.486 ± 0.040	−6 ± 1
**6**	5.0	145 ± 60	146 ± 34 (73%)	10 ± 1 (27%)	0.339 ± 0.053	+43 ± 6
**7**	3.4	19 ± 4	16 ± 2 (74%)	201 ± 72 (26%)	0.346 ± 0.122	+48 ± 8

^1^ C (Pyrene) in CAC experiment = 1 µM, C (**5**–**7**) = 0.05 mM, 10 mM TRIS pH 7.4. ^2^ Size was measured at pH 2.0 using 10 mM Britton–Robinson buffer. ^3^ Size was measured at pH 10.0 using 10 mM Britton–Robinson buffer.

**Table 2:** Binding parameters of **5**–**7** with CT-DNA evaluated for the EthBr displacement experiment.

Macrocycle	*K_sv_* × 10^5^, M^−1^	R^2^	*K_q_* × 10^13^, M^−1^	*K_a_* × 10^5^, M^−1^	R^2^	*n*
**5**	0.85	0.98	0.85	0.11	0.99	0.74
**6**	1.29	0.99	1.29	0.17	0.99	0.83
**7**	2.29	0.99	2.29	5.59	0.99	1.17

## Data Availability

Data is contained within the article or supplementary material.

## Supplementary Materials

The following supporting information can be downloaded at: https://www.mdpi.com/article/10.3390/ijms232314889/s1.
