# Supplementary material for: Calix[4]arene Polyamine Triazoles: Synthesis, Aggregation and DNA Binding
Source: Int J Mol Sci. 2022 Nov 28;23(23):14889. doi: 10.3390/ijms232314889 (PMC9738031; doi:10.3390/ijms232314889)
Supplement: Supplementary file 1 [file ijms-23-14889-s001.zip › ijms-2029668-supplementary.pdf]

Supplementary Material

(a)

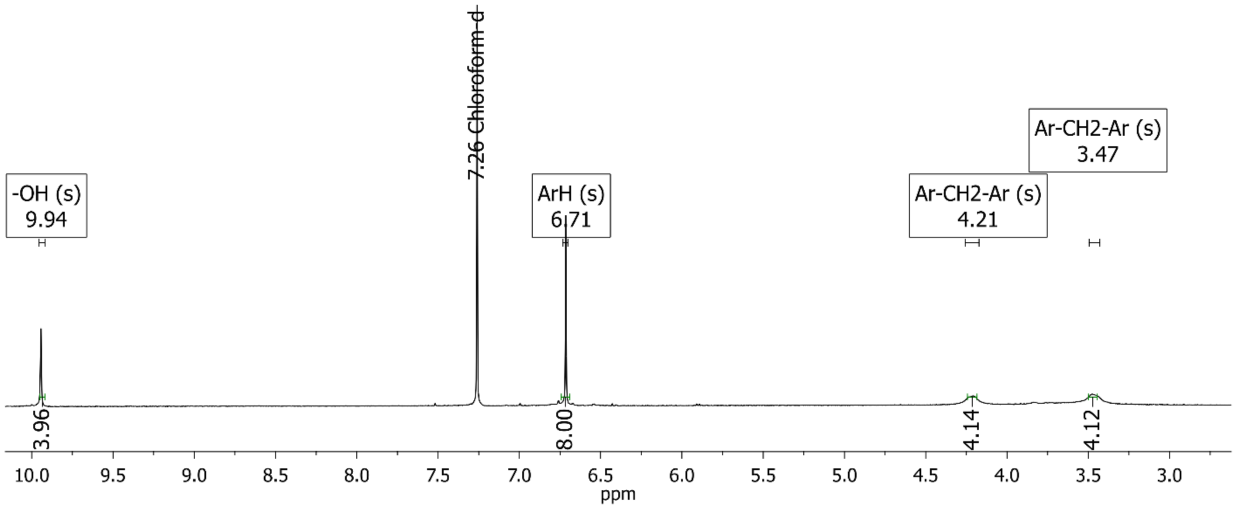

(b)

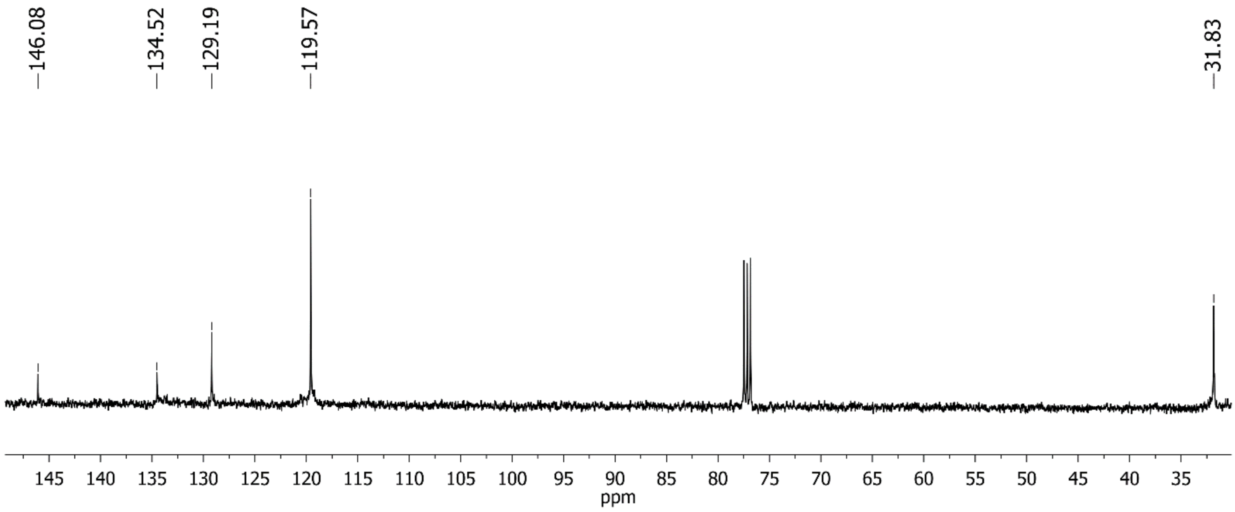

(c)

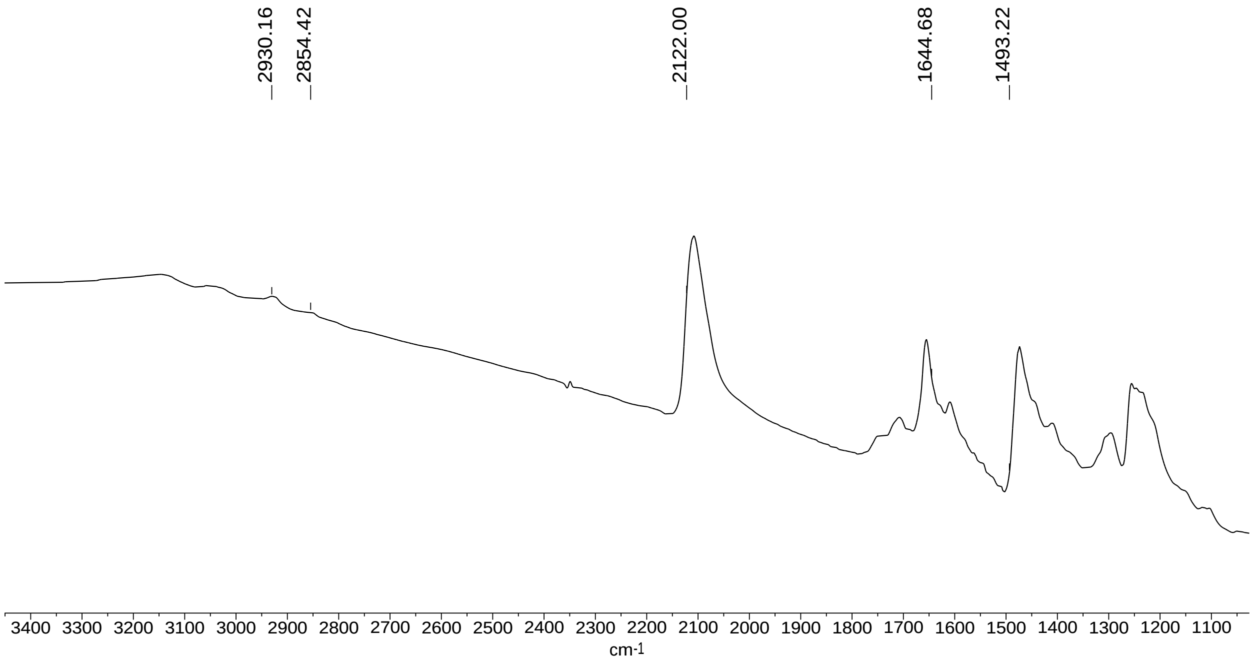

(d)

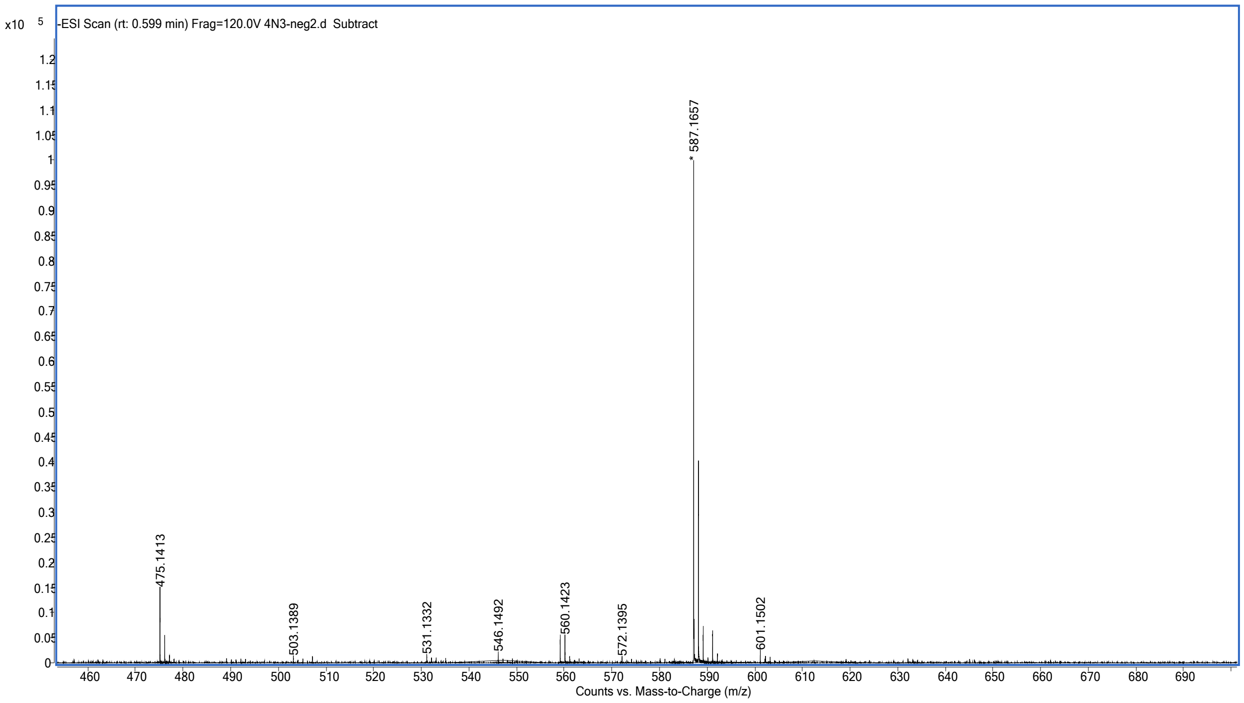

Figure S1. NMR ^{1}H (a), ^{13}C (b), FTIR (c) and HRESI MS (d) spectra of *5,11,17,23-tetraazide-25,26,27,28-tetrahydroxycalix[4]arene* **3**.

(a)

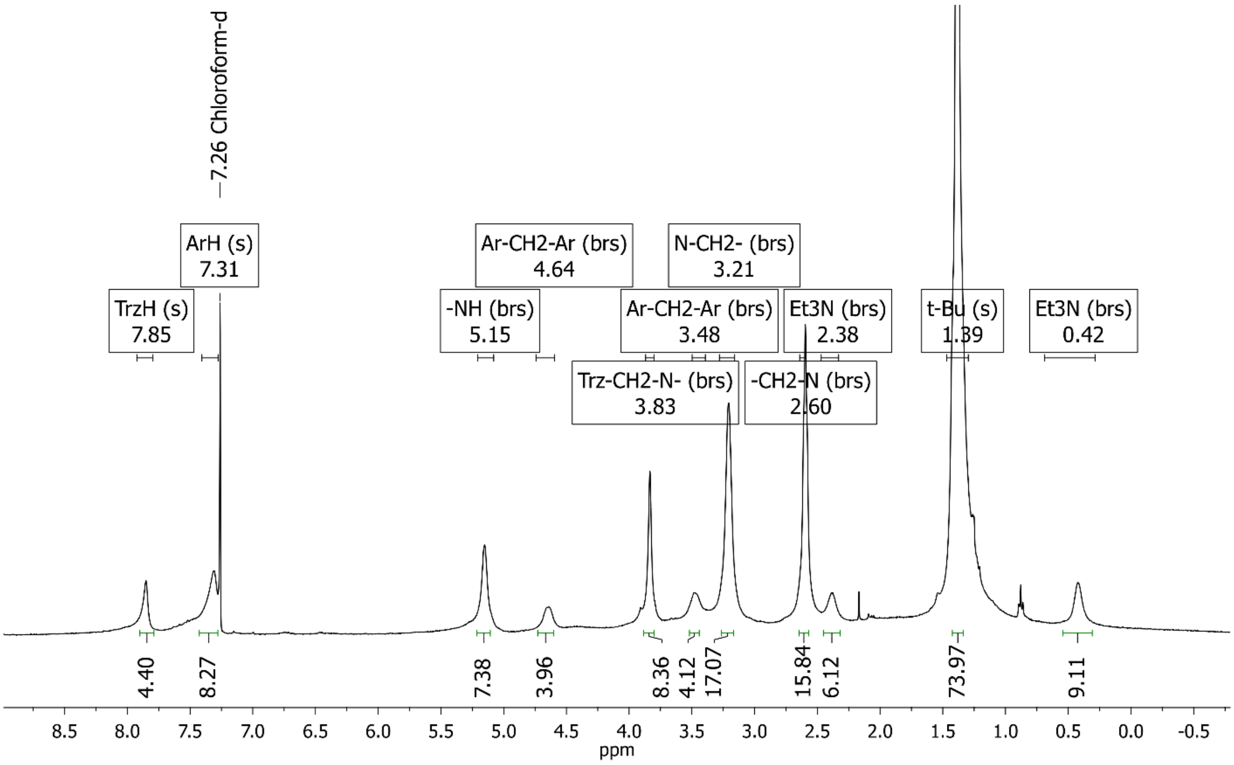
7.26 Chloroform-d
ArH (s)
7.31
TrzH (s)
7.85
Ar-CH2-Ar (brs)
4.64
-NH (brs)
5.15
N-CH2- (brs)
3.21
Ar-CH2-Ar (brs)
3.48
Trz-CH2-N- (brs)
3.83
Et3N (brs)
2.38
-CH2-N (brs)
2.60
t-Bu (s)
1.39
Et3N (brs)
0.42
4.40
8.27
7.38
3.96
8.36
4.12
17.07
15.84
6.12
73.97
9.11
ppm

(b)

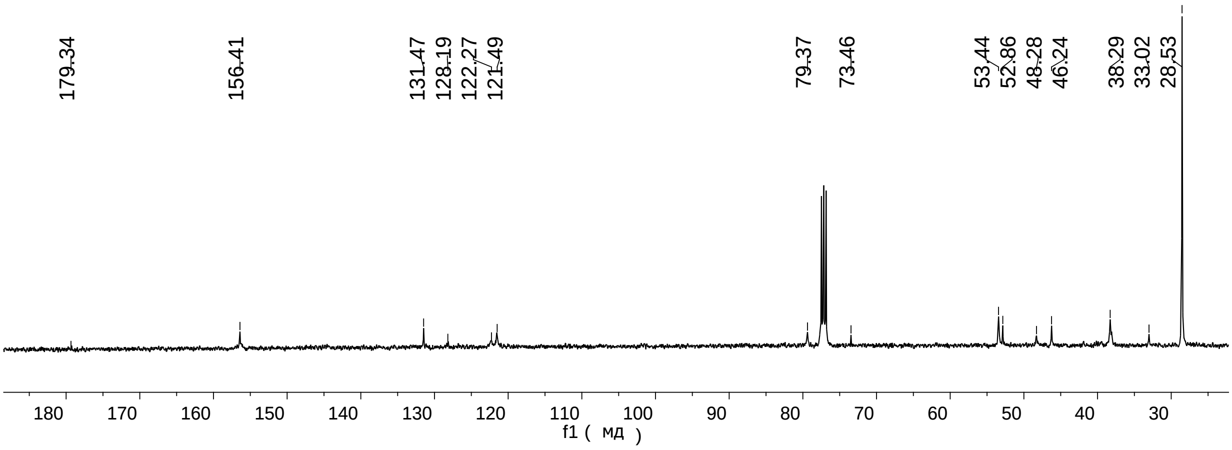
179.34
156.41
131.47
128.19
122.27
121.49
79.37
73.46
53.44
52.86
48.28
46.24
38.29
33.02
28.53
f1 (мд)

(c)

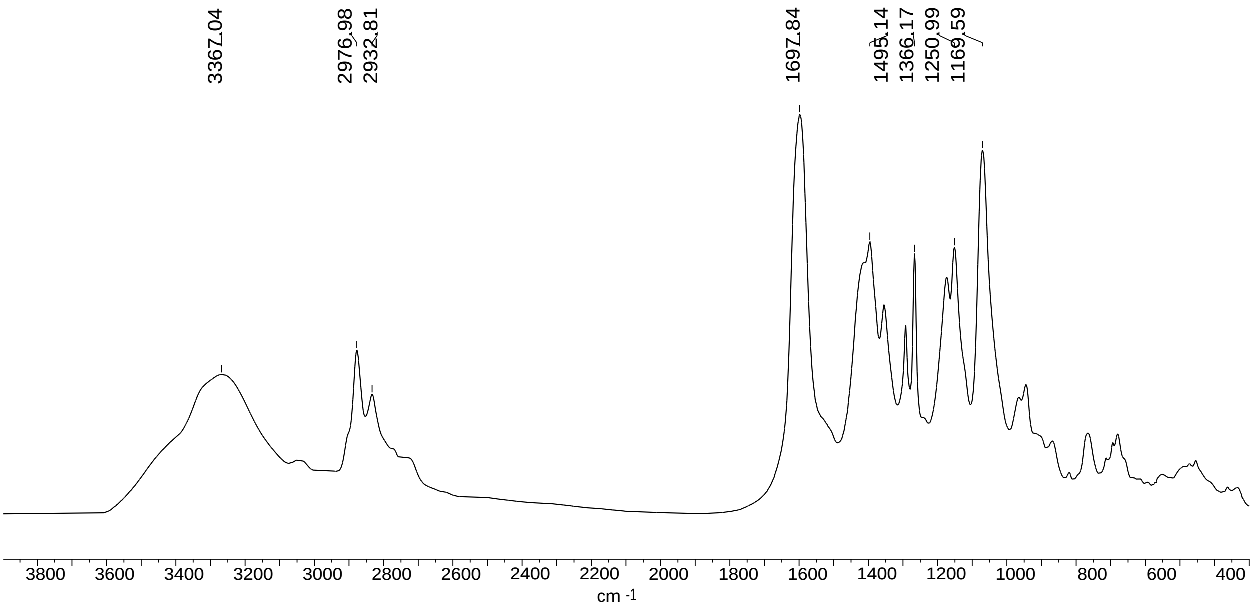

(d)

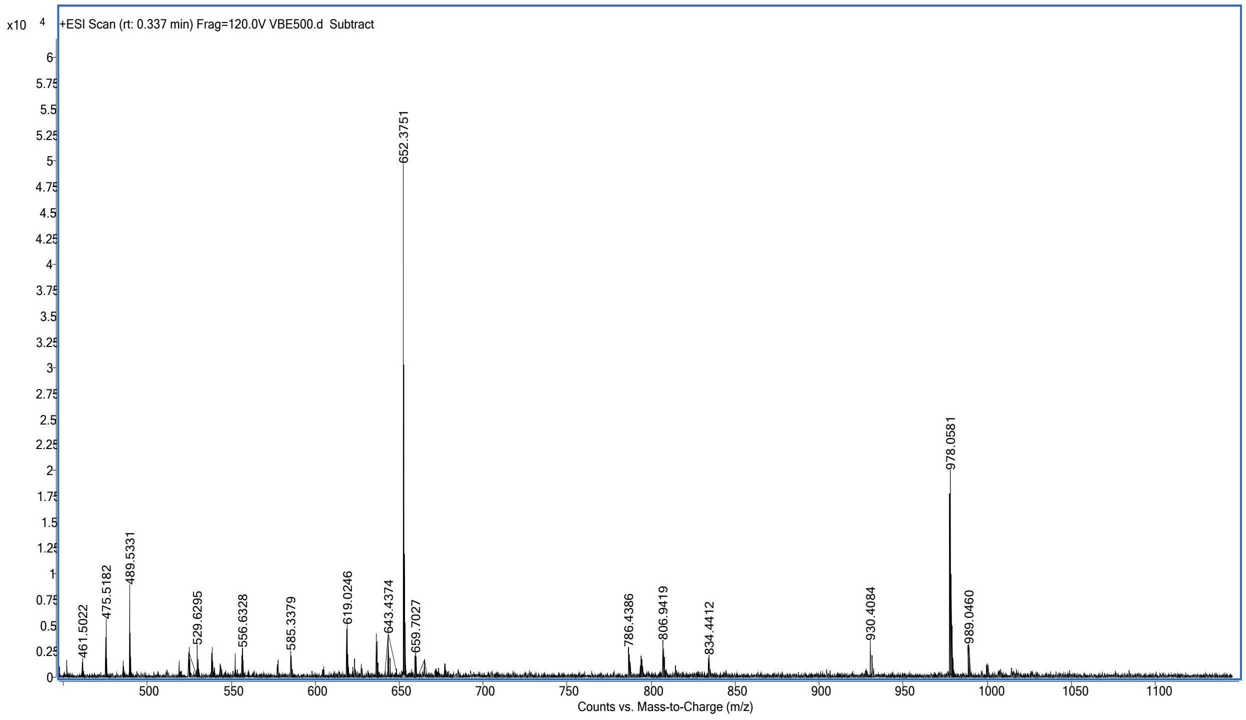

Figure S2. NMR ^{1}H (a), ^{13}C (b), FT IR (c) and HRESI MS (d) spectra of *(5,11,17,23-tetra(4-((bis(2-((tert-butoxycarbonyl)amino)ethyl)amino)methyl)-1H1,2,3-triazol-1-yl))-25,26,27,28-tetrahydroxycalix[4]arene)* **4**.

a)

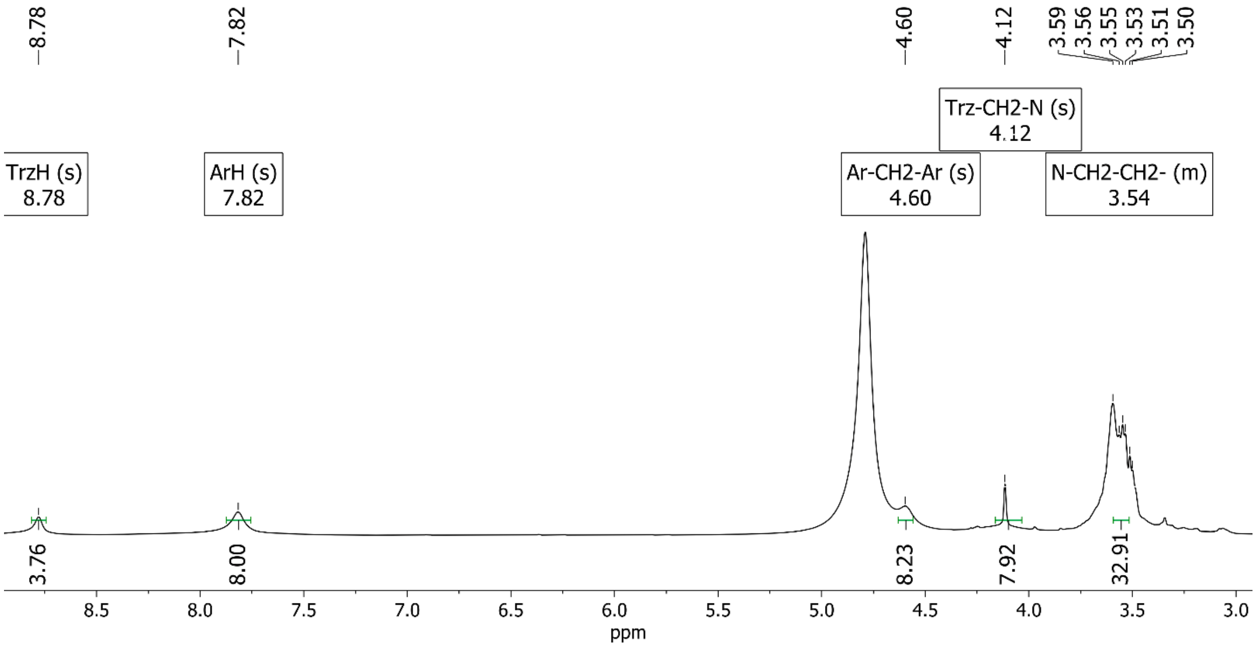
8.78
7.82
4.60
4.12
3.59
3.56
3.55
3.53
3.51
3.50
Trz-CH2-N (s)
4.12
TrzH (s)
8.78
ArH (s)
7.82
Ar-CH2-Ar (s)
4.60
N-CH2-CH2- (m)
3.54
3.76
8.00
8.23
7.92
32.91
8.5
8.0
7.5
7.0
6.5
6.0
5.5
5.0
4.5
4.0
3.5
3.0
ppm

b)

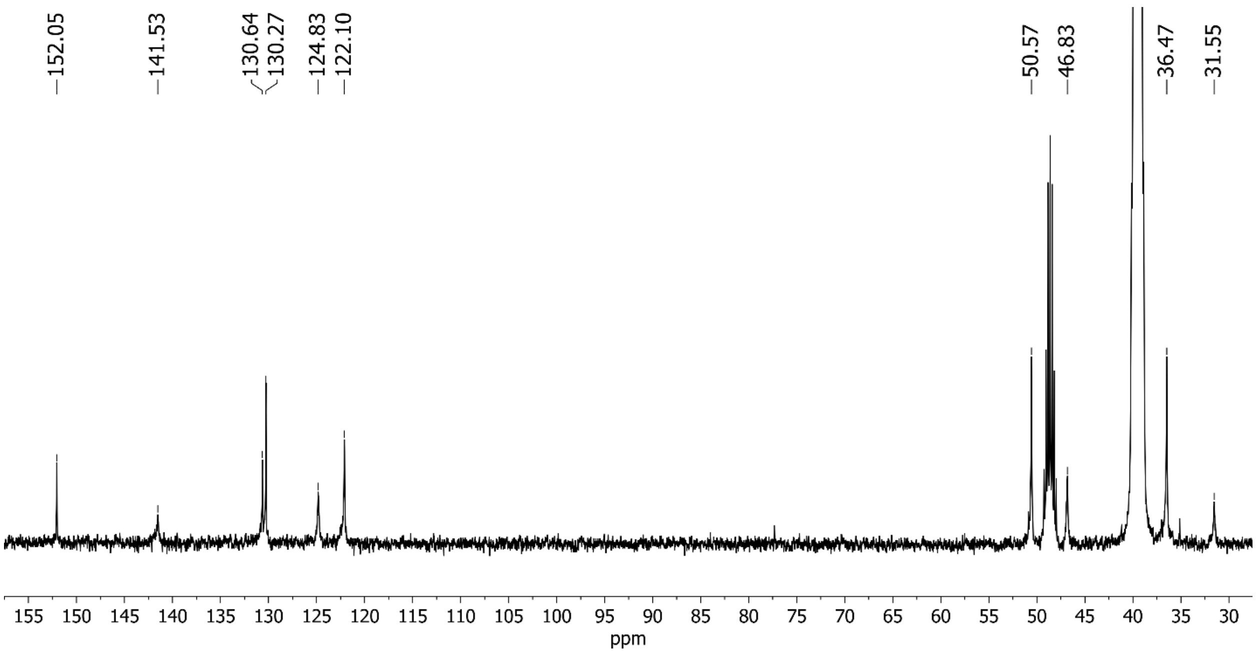
152.05
141.53
130.64
130.27
124.83
122.10
50.57
46.83
36.47
31.55
155
150
145
140
135
130
125
120
115
110
105
100
95
90
85
80
75
70
65
60
55
50
45
40
35
30
ppm

(c)

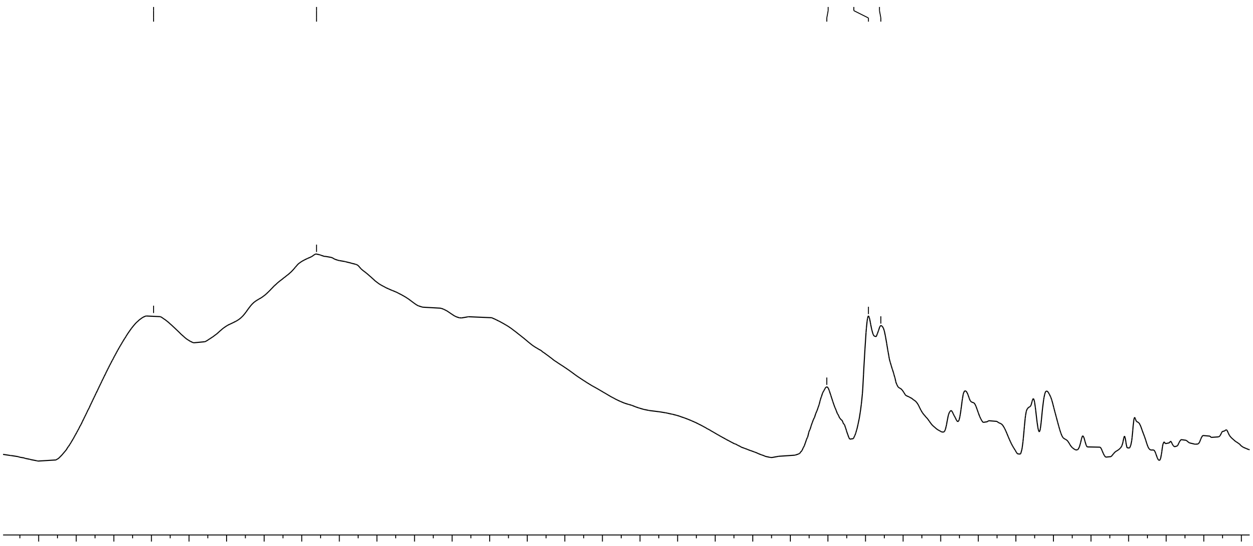

(d)

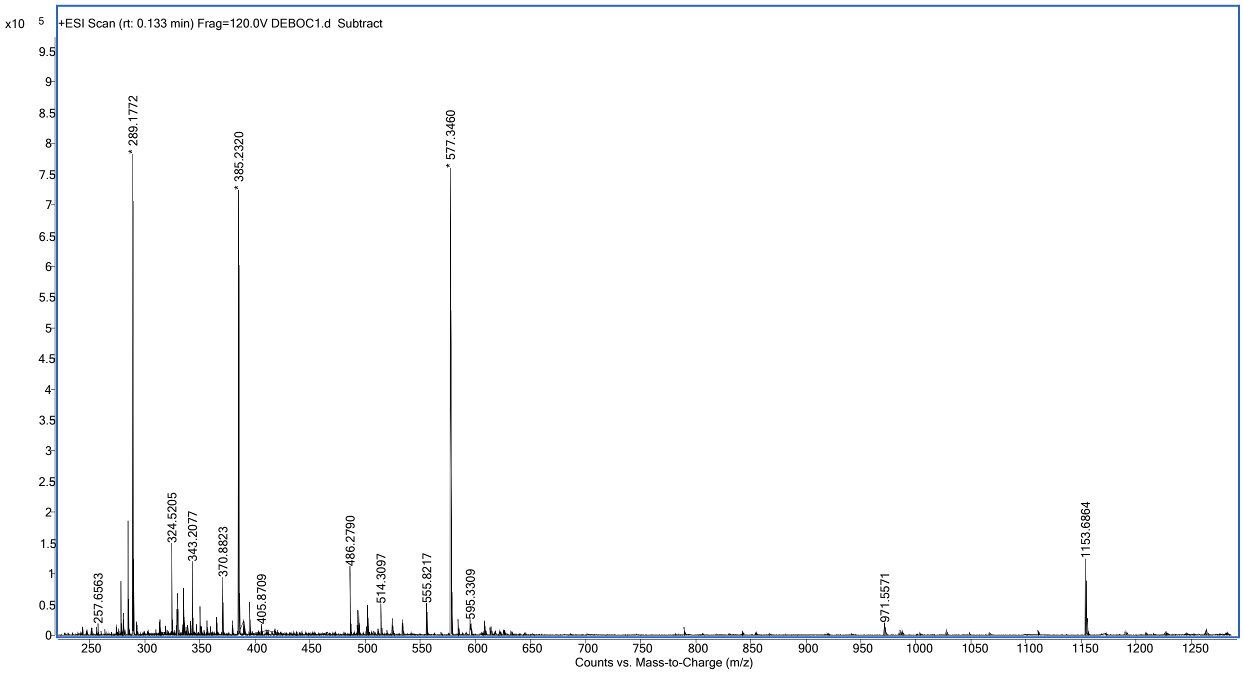

Figure S3. NMR ^{1}H (a), ^{13}C (b), FTIR (c) and ESI (d) spectra of *(5,11,17,23-tetra(4-((bis(2-(amino)ethyl) amino)methyl)-1H-1,2,3-triazol-1-yl))- 25,26,27,28-tetrahydroxycalix[4]arene octahydrochloride)***5**.

(a)

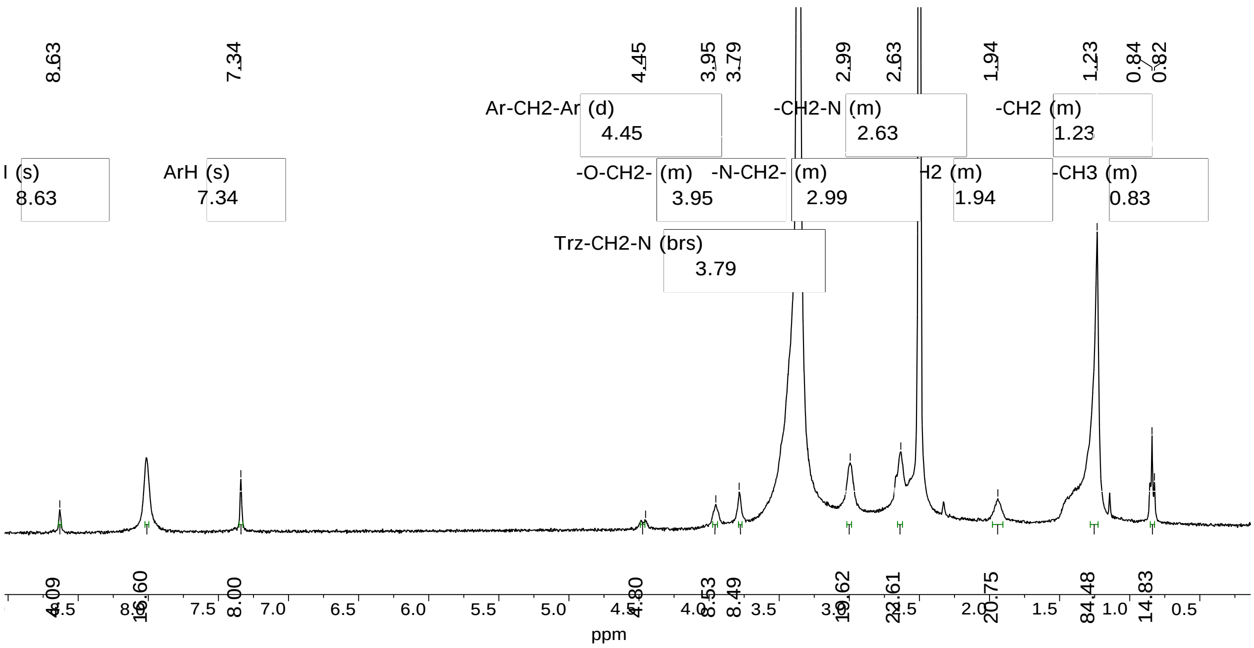

Ar-CH2-Ar (d)
4.45
-CH2-N (m)
2.63
-CH2 (m)
1.23
ArH (s)
7.34
-O-CH2- (m)
3.95
-N-CH2- (m)
2.99
-CH3 (m)
0.83
Trz-CH2-N (brs)
3.79
ppm

(b)

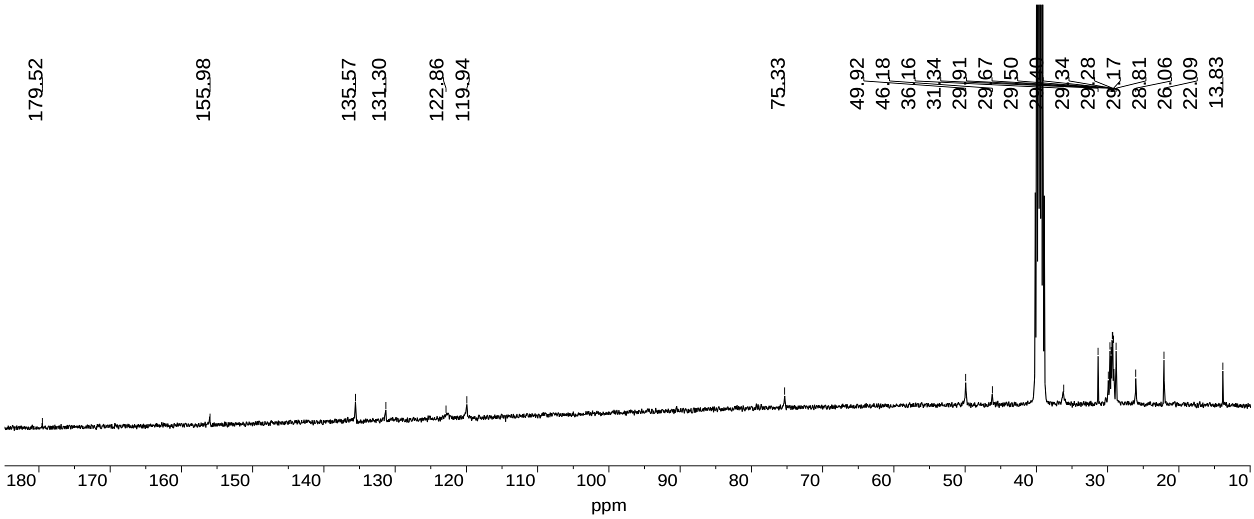

179.52
155.98
135.57
131.30
122.86
119.94
75.33
49.92
13.83
ppm

(c)

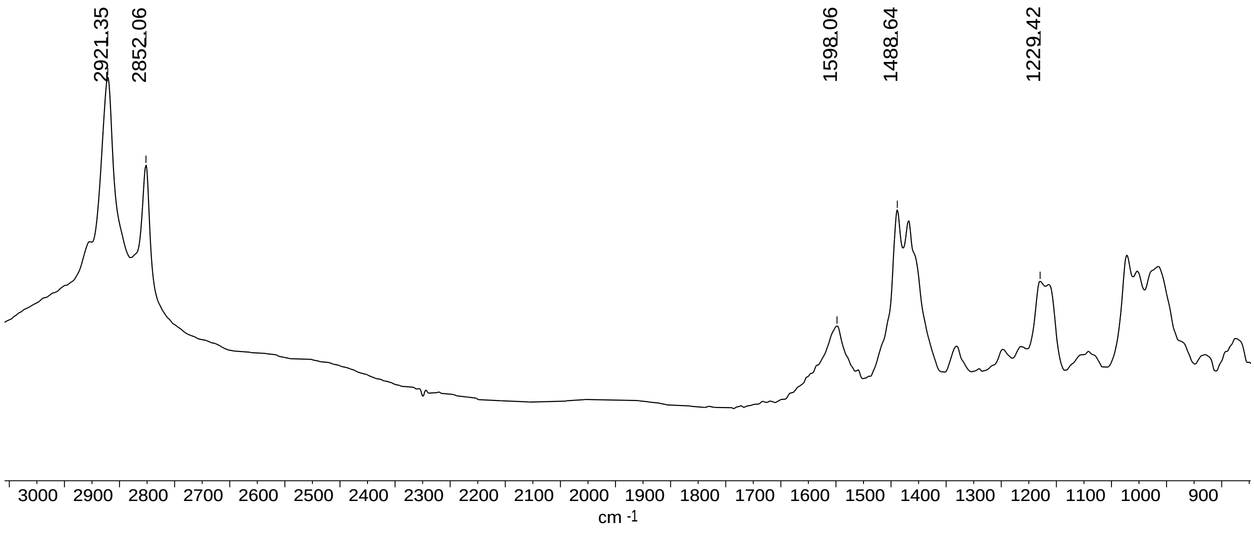

(d)

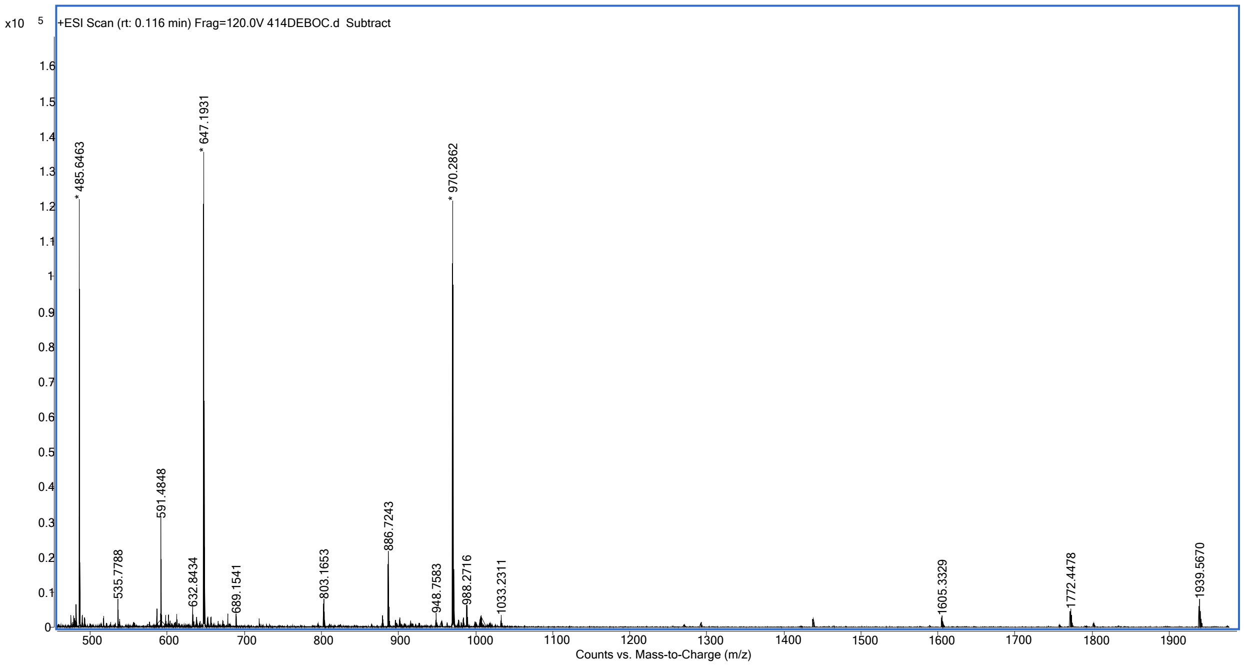

Figure S4. NMR ^{1}H (a), ^{13}C (b), FTIR (c) and ESI (d) spectra of *(5,11,17,23-tetra(4-((bis(2-(amino)ethyl) amino)methyl)-1H-1,2,3-triazol-1-yl))- 25,26,27,28-tetratetradecylcalix[4]arene octahydrochloride)* 7.

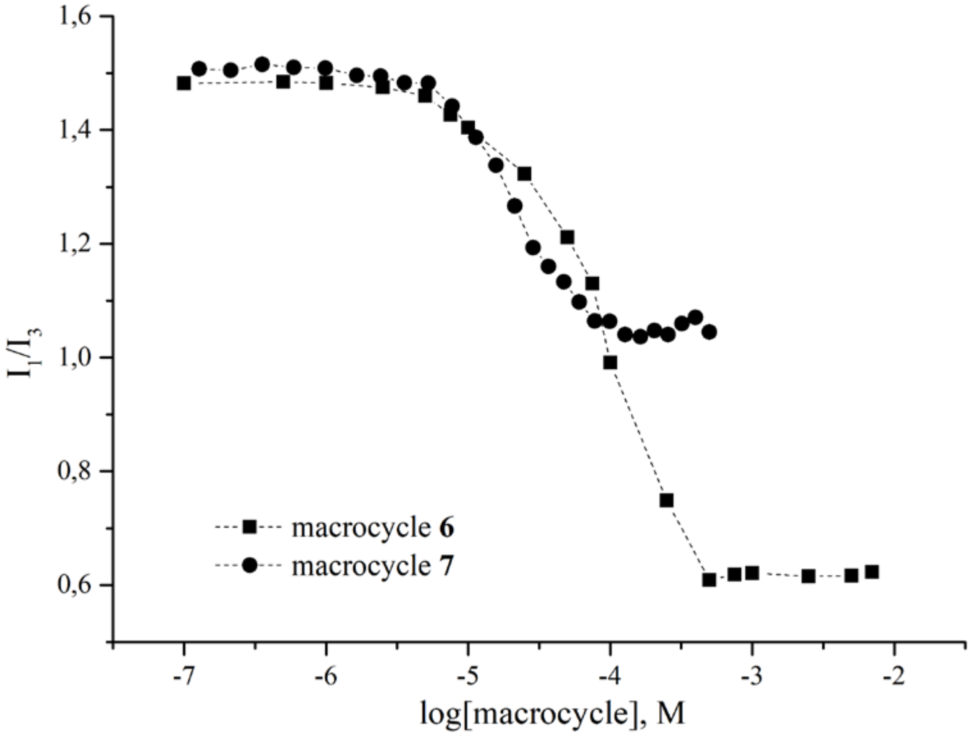

Figure S5. Dependence of the ratio of the first and third pyrene emission peaks on the logarithm of the concentration of macrocycles **6**, **7**.

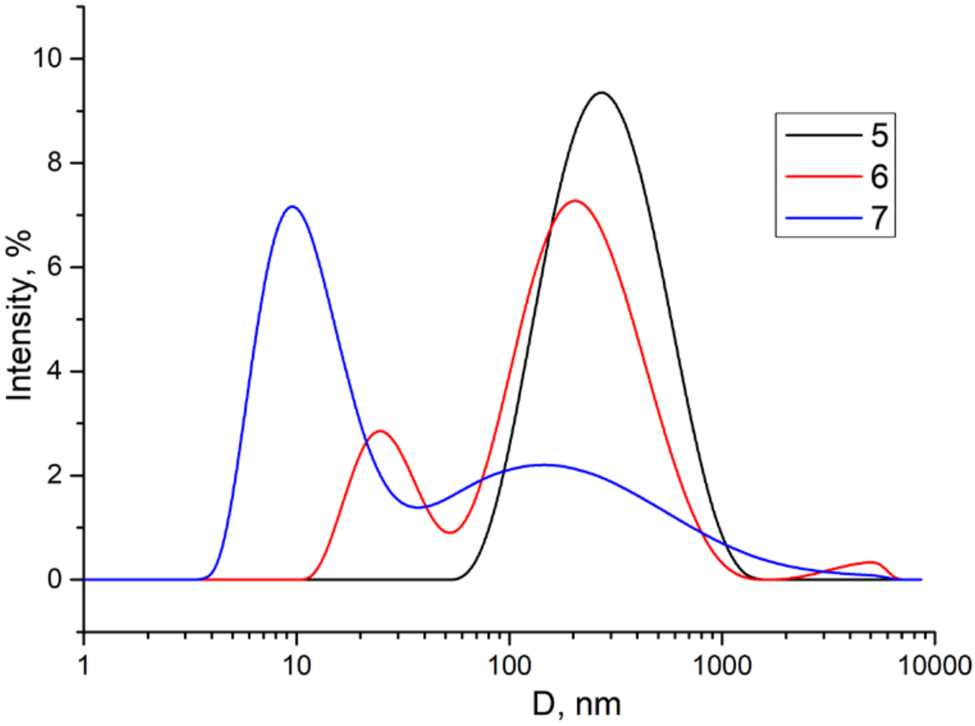

Figure S6. DLS intensity vs D graph for solutions of **5-7** (C = 0.05 mM, 10 mM TRIS).

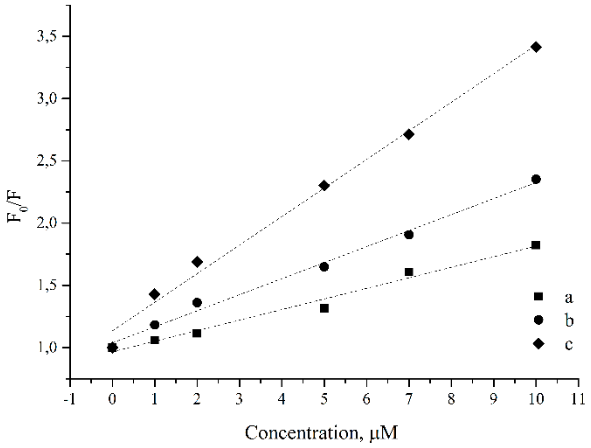

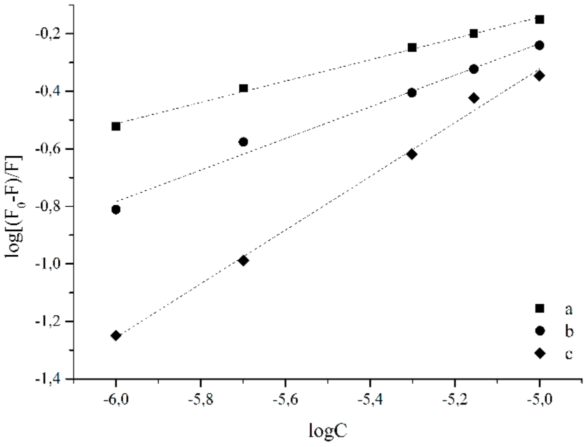

(A) (B)

Figure S7. (A) Stern-Volmer plot of CT-DNA titrated with macrocycles **5-7**. (B) The plot of -log ((F_0-F)/F) *vs* -log[macrocycle]. a – **5**, b – **6**, c – **7**.

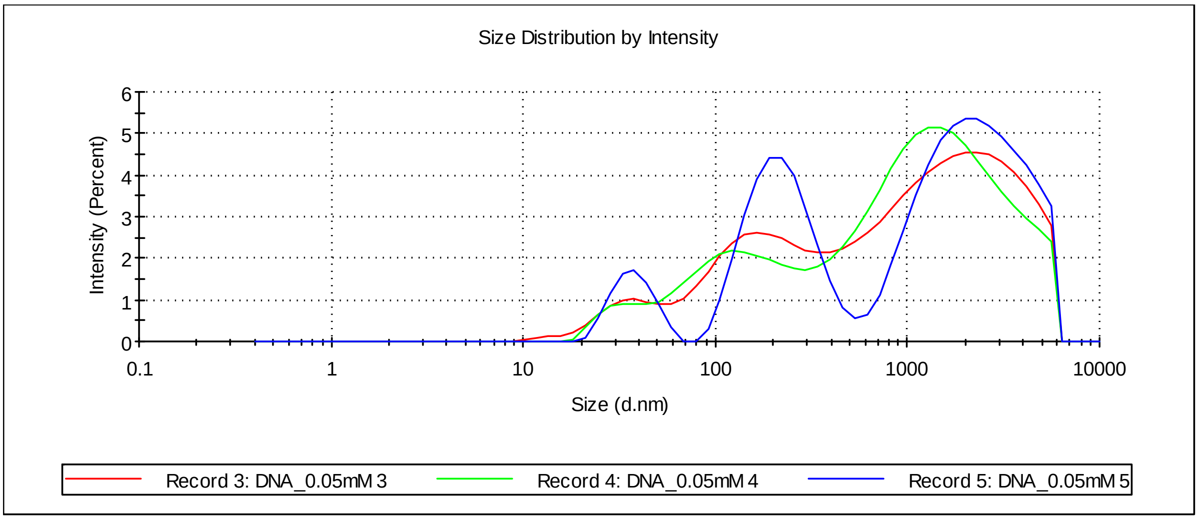

(A)

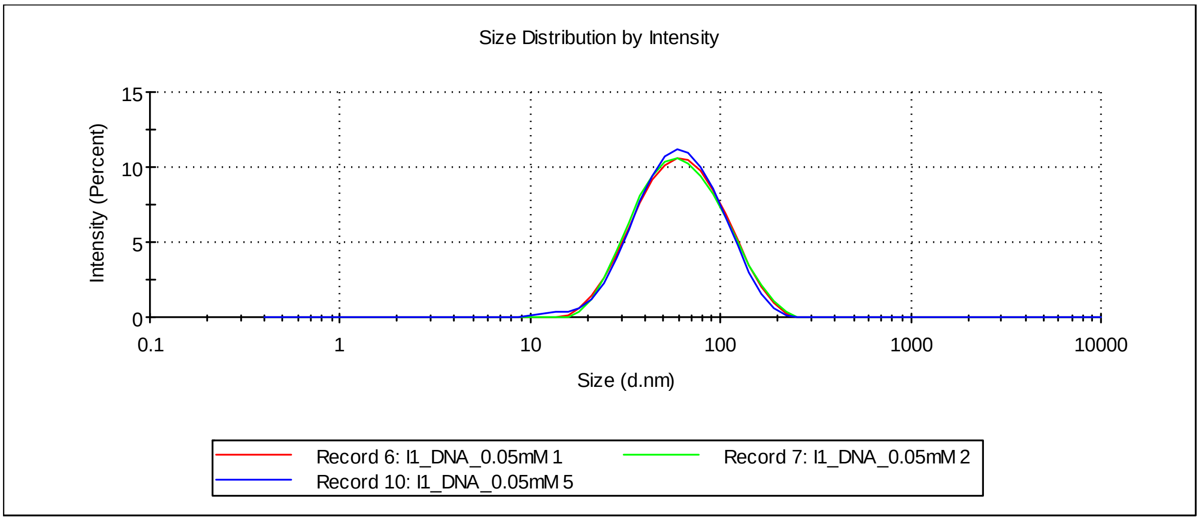

(B)

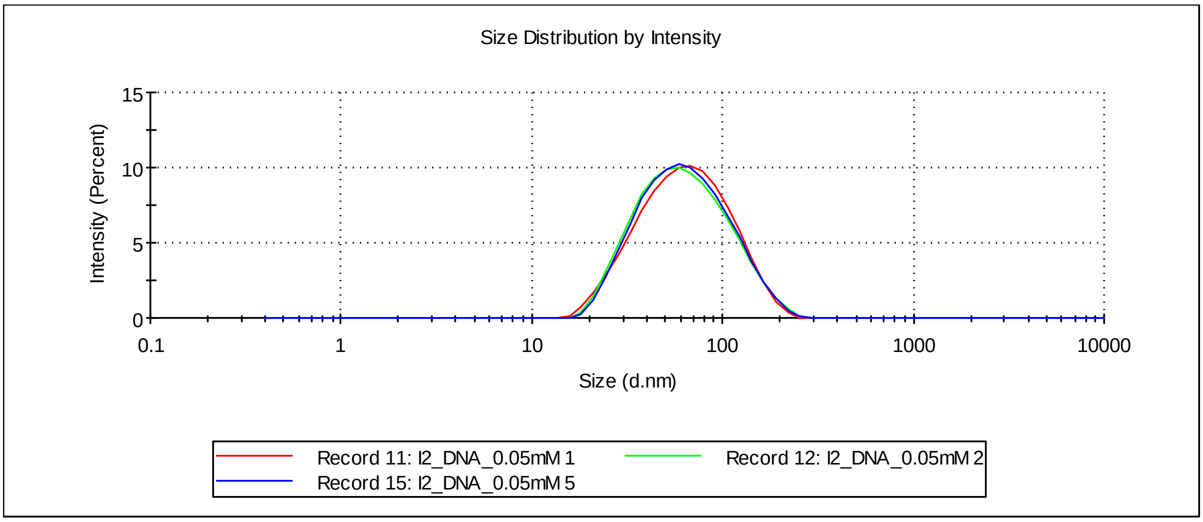

(C)

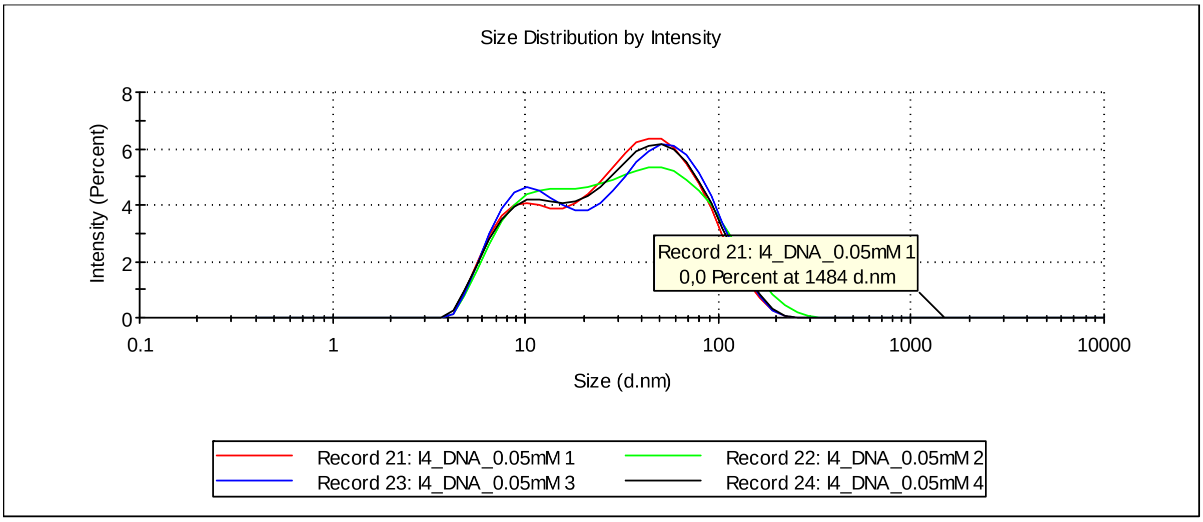

(D)

Figure S8. DLS intensity *vs* diameter graph for solutions of CT DNA (A) and CT-DA with **5** (B), **6**(C) or **7**(D) (C [CT DNA]= 0.05 mM, C [**5-7**] = 0.1 mM,10 mM TRIS).
